# Robust machine-learning based prognostic index using fatty acid metabolism genes predicts prognosis and therapy responses in glioblastoma

**DOI:** 10.7150/jca.117209

**Published:** 2025-08-22

**Authors:** Erjie Zhao, Zihan Wang, Liya Tang, Longxiu Zhang, Liuguijie He, Mengdie Li, Xin Ge, Zhumei Shi, Xu Qian, Risheng Cao

**Affiliations:** 1 Department of Nutrition and Food Hygiene, Key Laboratory of Public Health Safety and Emergency Prevention and Control Technology of Higher Education Institutions in Jiangsu Province, School of Public Health, Nanjing Medical University, Nanjing 210029, China.; 2 Department of Neurology, The First Medical Center of Chinese PLA General Hospital, Beijing 100853, China.; 3 Department of Neurosurgery, The First Affiliated Hospital of Nanjing Medical University, Nanjing, Jiangsu 210029, China.; 4 Key Laboratory of Human Functional Genomics of Jiangsu Province, Nanjing Medical University, Nanjing 210029, China.; 5 Department of Science and Technology, The First Affiliated Hospital of Nanjing Medical University, Nanjing, Jiangsu 210029, China.

**Keywords:** fatty acid metabolism, glioblastoma, machine learning, prognosis, precision oncology

## Abstract

**Background**: Glioblastoma (GBM) is the most prevalent and aggressive type of primary brain tumor in adults. Fatty acid metabolism plays a crucial role in promoting tumorigenesis, disease progression, and therapeutic resistance through the regulation of lipid synthesis, storage, and catabolism. However, its potential for predicting both prognosis and treatment response in glioblastoma is unexplored.

**Methods**: We systematically compiled fatty acid metabolism-related genes (FAMGs) from published literature and databases. A fatty acid metabolism signature (FAMS) was developed using a machine learning-based framework. The predictive performance of the FAMS was rigorously validated across multiple independent cohorts. Additionally, we investigated the associations between FAMS and clinical characteristics, mutation profiles, tumor microenvironment features, and biological functions.

**Results**: Our analysis revealed distinct FAMGs expression patterns in patients with GBM, which correlated with varying survival outcomes. Leveraging a robust machine learning framework, we established a fatty acid metabolism-based prognostic model. The FAMS emerged as an independent predictor of overall survival and other survival endpoints. Patients with lower FAMS exhibited enrichment in mitosis- and DNA repair-related pathways, which is linked to better survival. Conversely, higher FAMS scores were associated with enhanced immune activation, cellular proliferation, and chemotaxis, suggesting a greater likelihood of benefitting from immunotherapy.

**Conclusion**: We developed a reliable fatty acid metabolism signature capable of stratifying GBM patients on the basis of prognosis. The FAMS serves as an independent prognostic indicator and may offer clinical utility in guiding personalized treatment strategies for patients with GBM.

## Introduction

Glioblastoma (GBM) is the most prevalent and aggressive type of primary brain tumor in adults. Patients have limited treatment options and have worse survival globally 1-3. Although advancements in treatment, such as chemotherapy, surgical resection and even immunotherapy, have been made, the survival outlook of GBM patients remains poor. This can largely be attributed to the extensive heterogeneity of tumors and their ability to evade immune surveillance 4, 5. Many efforts have been made to understand the metabolic foundations of GBM and find new therapeutic targets, but the results are limited 6-8. Therefore, GBM is still a challenging medical problem. More in-depth research is needed to improve treatment outcomes.

Fatty acid (FA) metabolism is important for energy production and storage. Many biological processes, including cell proliferation and the generation of signaling molecules, depend on fatty acids. Recently, more attention has been given to its pivotal role in cancer 9-11. In the central nervous system (CNS), FAs are particularly important because the majority of the dry mass of the brain is composed of lipids 12. In addition, many CNS processes require FAs, such as the generation of myelin 13, the growth and regeneration of axons 14, and the transport of neurotransmitters 15. Researchers have reported that FA metabolism is involved in tumor cell proliferation, metastasis, and even treatment resistance in brain tumors 16-20 through different mechanisms. In addition, the metabolic patterns of tumor cells differ markedly from those of normal cells, and these differences may affect the local metabolic landscape and mediate antitumor immunity 21. Lipid synthesis and metabolic signaling can promote the antitumor function of regulatory T-cells (Tregs) in the tumor microenvironment 22. These tumor-associated macrophage (TAM) subpopulations, termed lipid-laden macrophages, intersect with mesenchymal-like (MES-like) GBM cells to promote the malignant transformation of tumors and immunosuppression 23. Hence, targeting fatty acid metabolism represents a potential approach for the treatment of GBM. In light of current studies focused on the transcriptomic signatures associated with fatty acid metabolism in GBM, we aimed to quantify a fatty acid metabolism signature at the transcriptomic level, which can enhance risk stratification and provide guidance for treatment of GBM.

In this research, we first characterized the status of fatty acid metabolism genes (FAMGs) in GBM and established a robust Fatty Acid Metabolism Prognostic Signature (FAMS) by integrating multiple machine learning survival algorithms. We validated the feasibility of FAMS in both training and validation cohorts from different data platforms and classified all GBM patients into high- and low-risk groups. Additionally, we explored the underlying relationships between FAMS and biological function and immune cell infiltration in the TME. Our analysis highlights the importance of the FAMS in predicting the prognosis and response to treatment in patients with GBM.

## Materials and Methods

### Data collection and processing

RNA-seq data, somatic mutation and copy number variation (CNV) data, and corresponding meta-information for the TCGA-GBM cohort 24 were obtained from The Cancer Genome Atlas (TCGA) database using TCGAbiolinks (v2.32.0) 25. The raw microarray data of the TCGA-GBM cohort 26 were downloaded from GDC (https://portal.gdc.cancer.gov/). The transcriptomic data of the normal brain cortex, along with other relevant information, were retrieved from the Genotype-Tissue Expression (GTEx v7, https://www.gtexportal.org/home/datasets) database 27. In addition, the data from the other two CGGA batches, mRNAseq_693 28 and mRNAseq_325 29 were downloaded from the Chinese Glioma Genome Atlas (CGGA, http://www.cgga.org.cn/index.jsp). All the RNA-seq data were mapped to the human reference genome hg19, and we retained genes that appeared in all the data for subsequent analysis. Another two microarray datasets, GSE16011 30 and GSE13041 31, were acquired from GEO (http://www.ncbi.nlm.nih.gov/geo).

We retained all GBM patients whose overall survival (OS) data for further analysis. The raw read counts from the TCGA and CGGA cohorts were converted to transcripts per kilobase million (TPM) and further log2 transformed. The raw microarray data were background adjusted and normalized via the robust multiarray averaging (RMA) algorithm 32. We used the “maftools” package (v2.20.0) to analyze the somatic mutation and CNV data 33.

To collect the FAMGs, we obtained gene sets related to fatty acid metabolism from the Kyoto Encyclopedia of Genes and Genomes (KEGG, v111.1) 34 and the Molecular Signatures Database (MsigDB, v2024.1. Hs) 35 hallmark and Reactome (v88) 36 database. In conclusion, 332 fatty acid metabolism genes were identified following the exclusion of overlapping genes from the previously mentioned data source.

### Genetic alterations and differential expression analysis of FAMGs in glioblastoma

The top genes with the highest mutation frequency are shown by oncoplot. The frequency of CNVs in FMGs was subsequently assessed, and the most significant findings were represented using a bidirectional lollipop chart for visualization. KEGG enrichment analysis was conducted using the R package “ClusterProfiler” (v4.12.6) 37. To identify the differentially expressed genes (DEGs) among the FAMGs, we utilized data from the normal brain cortex data as a reference. The DESeq2 (v1.44.0) package in R was employed to determine DEGs, applying the significance criteria of |log2FC| > 1 and an adjusted p value of less than 0.05 38.

### Consensus clustering of FAMGs

Utilizing the RNA expression of the differentially expressed FAMGs, we performed consensus clustering using the “ConsensusClusterPlus” (v1.68.0) R package 39. Next, we integrated the consensus score matrix, the cumulative distribution function (CDF) curve, and the proportion of ambiguous clustering (PAC) score to determine the optimal number of clusters. These metrics collectively provide a robust framework for selecting the most appropriate clustering solution 40. To gain deeper insights into the functions of the expression clusters, we conducted GO and KEGG analyses. In addition, single-sample gene set enrichment analysis (ssGSEA) 41 was used to characterize the differences between cancer hallmark functions and immune cell proportions.

### Calculation of FAMS by machine learning

To establish an FAMGs-based prognostic signature, we first screened the prognostic FAMGs by univariate Cox regression analysis and integrated 10 machine learning algorithms to construct a prediction model. Finally, all combinations of these algorithms were performed using the ten-fold cross-validation method in TCGA-GBM mRNA-seq data 24 to train the model. All the models were evaluated using five additional validation datasets from different data platforms, including TCGA microarray 26, CGGA mRNA sequencing (mRNAseq_693 and mRNAseq_325) 28, 29, GSE16011^30^, and GSE13041^31^. To select the model with the best performance, the Harrell's concordance index (C-index) 42 was calculated across the five validation datasets. An average C-index was used to select the final model to construct a fatty acid metabolism prognostic signature (FAMS).

The median value of the FAMS was used to stratify patients into high- and low-risk groups; the prognostic and predictive role of the FAMS were explored on the basis of this situation. Survival analyses were then performed for these two groups across all GBM patients or those receiving treatment. Multivariate Cox regression analysis was carried out to assess the independence of FAMS from other clinical factors.

### Gene set enrichment analysis (GSEA)

To fully describe the functional differences between different groups, we used GSEA 35 to explore signaling pathways and functions in GSEA software (v4.3.3). The log2-transformed fold changes of genes were imported into the software, and gene sets of hallmark genes and GO terms (BP and CC) obtained from the MSigDB database were set as background gene sets. After running the GSEA, we used Cytoscape (v3.10.2) 43 software and the EnrichmentMap (v3.4.0) 44 plug-in to visualize the functional landscape obtained from the GSEA results.

### Immune infiltration analysis

To characterize the immune response process, we employed the tracking tumor immune phenotype (TIP) webserver 45 to assess the cancer-immunity cycle. In addition, two tumor microenvironment-related signatures 46, 47 were collected and evaluated by the ssGSEA method.

The relative abundance of 28 immune cell types was also calculated by ssGSEA in each patient in the TCGA-GBM cohort with immune cell markers 48-50. The levels of immune checkpoint genes (such as CTLA4 and CD163) were compared and visualized. Spearman analyses were performed to assess the relationship between immune cell abundance and the calculated FAMS values.

### Statistical analysis

All the data were processed and statistically analyzed using R (v4.4.0) software. Kaplan-Meier (KM) survival analysis was conducted with the 'survminer' (v0.4.9) and 'survival' (v3.5-5) R packages 51-53. Differences in continuous variables between groups were evaluated using either the Wilcoxon rank sum test or the Student's t test depending on the data distribution, and the chi-square test was applied to compare categorical variables. The C-index of different models were computed using the 'Hmisc' package (v5.1-3) 54. All the statistical tests were two-sided. *p* < 0.05 was considered to indicate statistical significance.

## Results

### Genetic variant landscape and expression of FAMGs in GBM

**Figure 1:**
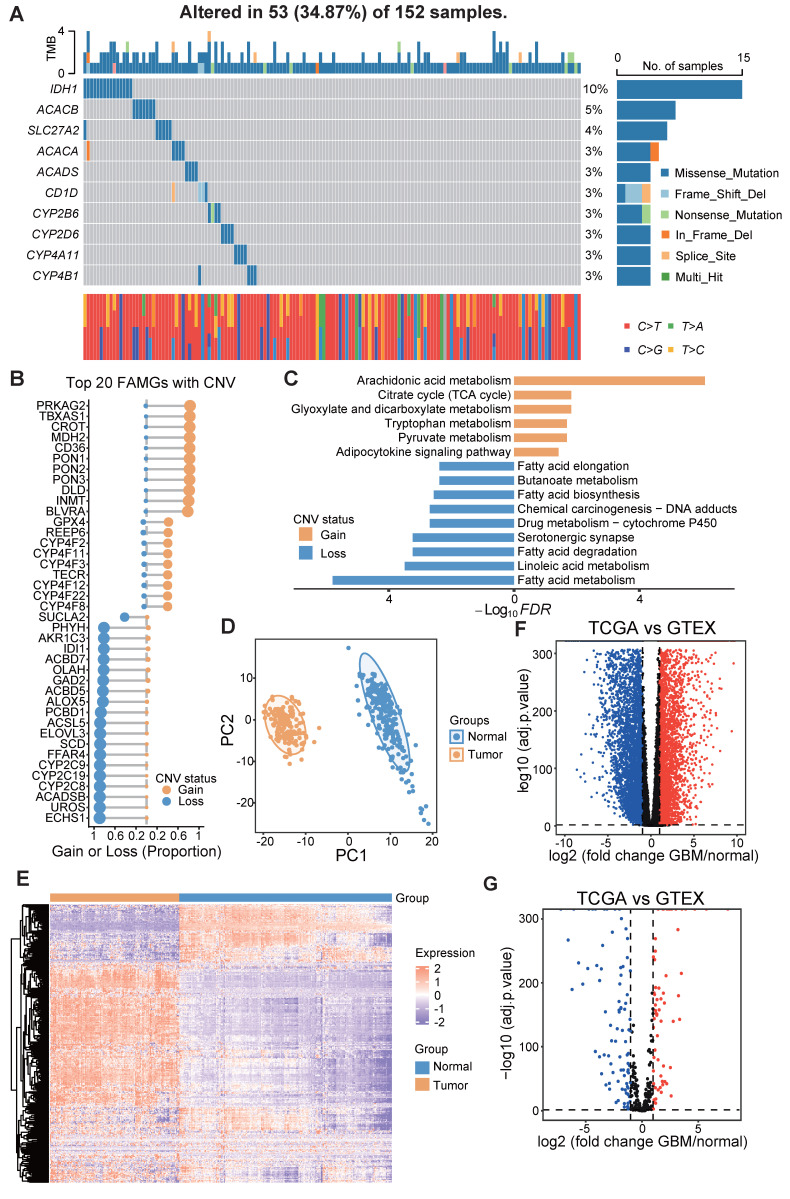
Genomics variation and expression of fatty acid metabolism related genes in GBM. **(A)** Mutation prevalence of top 10 FAMGs in GBM.** (B)** Copy number amplification or deletion frequency of top 20 FAMGs. **(C)** The KEGG analysis of the FAMGs with highest amplification or deletion frequency. **(D)** The PCA analysis based on FAMGs showed the heterogeneity between GBM patients and normal brain cortex. **(E)** The heatmap showed the expression pattern of FAMGs. **(F)** The volcano plot showed all differential expression genes between GBM and normal brain cortex tissue. **(G)** Volcano plot exhibited differential expression genes among FMGs.

A total of 332 FAMGs were curated from the KEGG, MSigDB, and Reactome databases (Table S1). First, we investigated the somatic mutation prevalence and CNV frequency of these fatty acid metabolism-related genes among GBM patients. Among them, *IDH1* had the highest mutation rate (up to 10%), and the mutation frequency of the other genes was relatively low (approximately 3%-5%) (Figure 1A). Copy number variation analysis revealed that many FAMGs were highly variable (Figure 1B). *PRKAG2*, *TBXAS1*, *CROT*, *MDH2*, and paraoxonase family genes (*PON1*, *PON2*, *PON3*) exhibited widespread CNV amplification, whereas *ECHS1*, *UROS*, *FFAR4*, and *SCD* exhibited CNV deletions. The FAMGs with high amplification frequency are involved primarily in arachidonic acid metabolism and the adipocytokine signaling pathway, and the FAMGs with high deletion frequency are associated with linoleic acid metabolism, fatty acid elongation and several other fatty acid metabolism-related biological processes (Figure 1C).

We examined the expression levels of these FAMGs in both glioblastoma and normal brain tissue. Principal component analysis (PCA) revealed a clear distinction between normal tissue and glioblastoma tissue on the basis of the expression patterns of FAMGs (Figure 1D). Similarly, it was obvious that expression of FAMGs exhibited significant differences between the normal and tumor groups (Figure 1E). Through differential expression analysis, we detected 152 FAMGs by comparing 166 glioblastoma samples with 255 normal brain cortex samples. Among the DEGs, 64 FAMGs were significantly upregulated in the samples from patients with GBM, whereas 88 FAMGs were notably downregulated in the samples from patients with GBM (Figure 1F, G).

### Consensus clustering of FAMGs in glioblastoma

**Figure 2:**
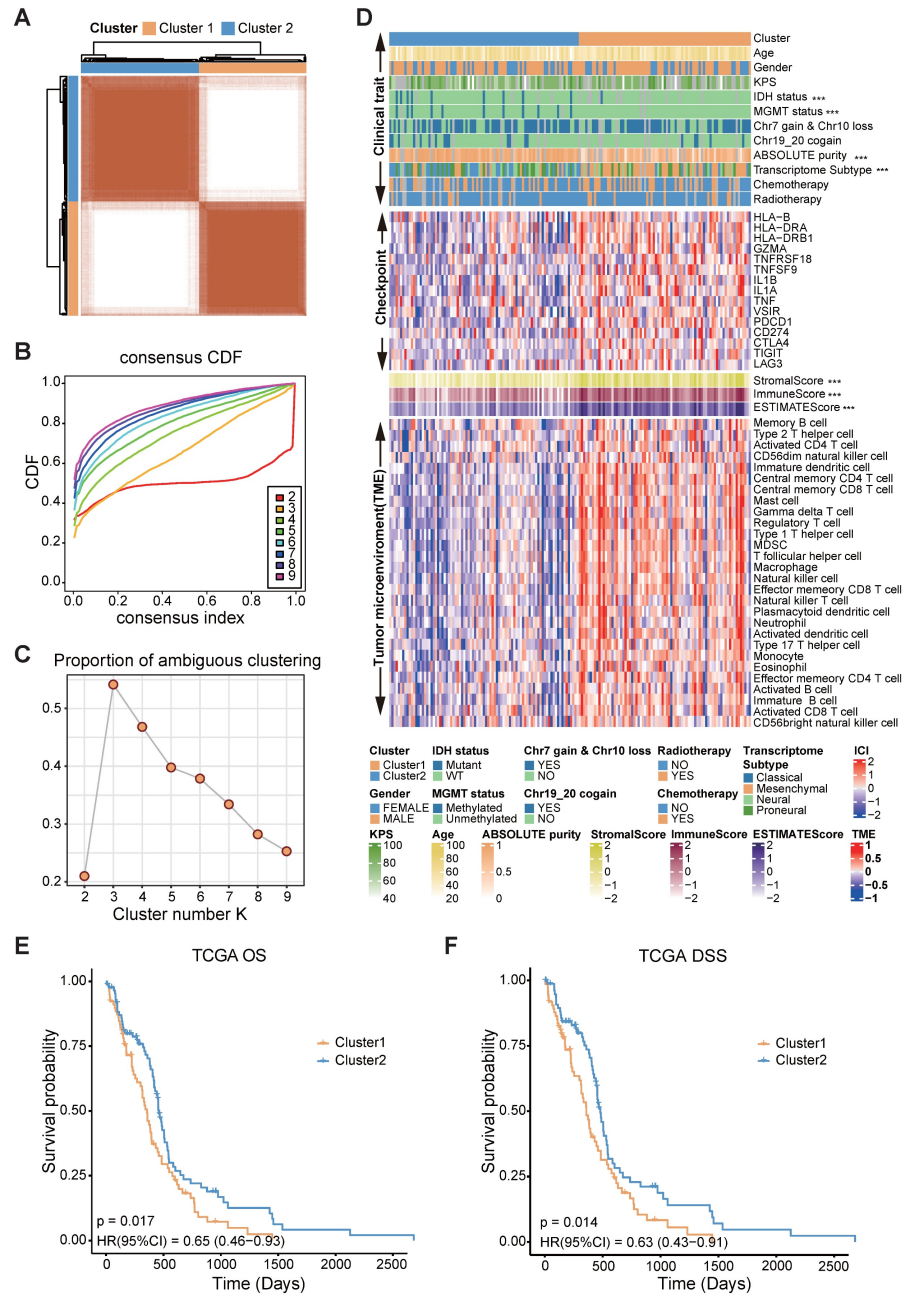
Correlation of FAMGs expression pattern with TME, stemness, and clinical traits. **(A)** Consensus score matrix of GBM mRNA-seq dataset from TCGA when k = 2. **(B)** The CDF curves with different k values (indicated by colors). **(C)** PAC score of different k values. **(D)** A heatmap showed the clinical traits, expression of immune checkpoint genes, immune score, stromal score and tumor microenvironment in TCGA GBM mRNA-seq dataset. The Wilcoxon rank sum test or the chi-square test was performed to assess the difference between the FAMGs cluster 1 and cluster 2. “****” represented that the p value < 0.05. **(E)** The Kaplan-Meier curves showed that overall survival (OS) of GBM patients in FAMGs cluster 1 and cluster 2. **(F)** The Kaplan-Meier curves showed the disease-specific survival (DSS) of GBM patients in FAMGs cluster 1 and cluster 2.

To underscore the clinical relevance of the FAMGs, we performed a consensus clustering analysis based on the differentially expressed FAMGs. The CDF curves and PAC statistics indicated that the patients could be divided into two FAMG patterns, named as cluster 1 and cluster 2 (Figure 2A-C, S1A, Table S2). Compared with cluster 2, cluster 1 had fewer samples with *IDH* mutation, *MGMT* promoter methylation, and chr 19/20 co-gain. In addition, the expression of the immune checkpoint genes *CD274*, *PDCD1*, *CTLA4*, *TNFRSF18*, *TNFSF9*, *TIGIT,* and *LAG* was high in cluster 1. Both the immune and stromal scores estimated by the ESTIMATE algorithm and the proportion of immune cells in the tumor microenvironment were significantly greater in cluster 1 (Figure 2D). The cancer hallmark function also showed the similar phenomena (Figure S1B). Moreover, the results of the KM analysis confirmed significant differences in overall survival (OS) and disease-specific survival (DSS) between the clusters (*p* = 0.017 and *p* = 0.014, respectively) (Figure 2E-F).

Furthermore, to gain insights into the molecular characteristics underlying this distinction, we identified 1024 genes that were differentially expressed between the clusters (Figure S1C). Functional enrichment analyses through GO, KEGG, and hallmark pathways revealed that the cluster 1 was closely related to immune responses: leukocyte chemotaxis and migration, hypoxia, and epithelial-to-mesenchymal transition (EMT) (Figure S2A-C). These findings indicate significant differences in biological functions between the two clusters categorized by FAMGs and demonstrate the rationality and implications of such categorization in glioblastoma.

### Establishment and validation of the fatty acid metabolism prognostic signature

**Figure 3:**
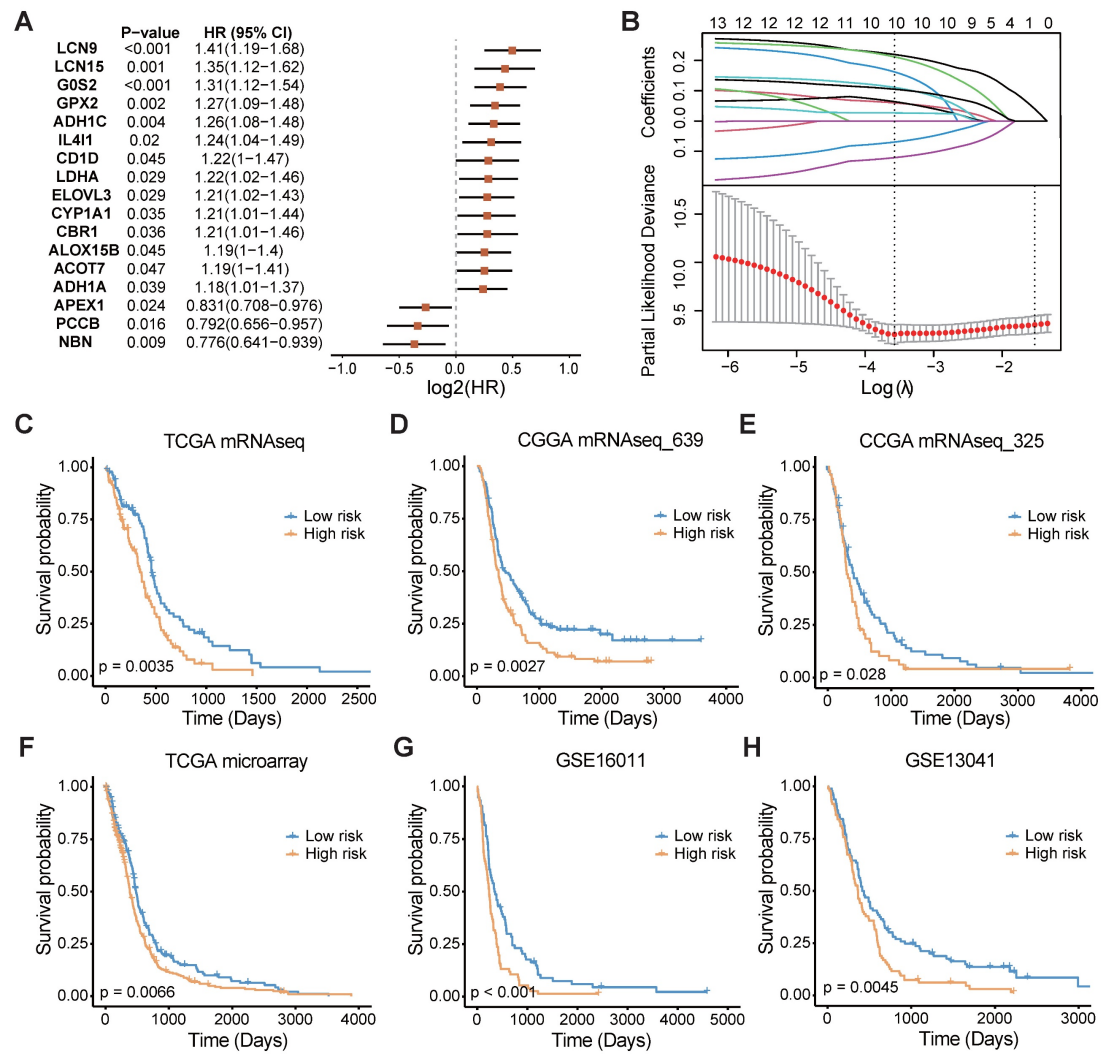
A consensus FAMS was developed and validated via the machine learning-based algorithms. **(A)** Univariate Cox analysis identified 17 prognostic FAMGs in the TCGA GBM mRNA-seq cohort. Data are presented as log2 hazard ratio (HR) ± 95% confidence interval [CI]. **(B)** The determination of the optimal λ in TCGA mRNA-seq data. **(C-E)** The Kaplan-Meier curves of OS according to the FAMS in TCGA mRNA-seq, CGGA mRNAseq_639, CGGA mRNAseq_325, TCGA microarray, GSE16011, GSE13041.

In light of the significant influence of FAMGs on the clinical outcomes and tumor environment of GBM patients, a FAMGs based prognostic signature was pursued to gain deeper insights into the underlying complexities of GBM. Through univariate Cox regression analysis, we initially identified prognosis-associated FAMGs among the differentially expressed FAMGs (Figure 3A). These FAMGs were analyzed using the machine learning method to develop a robust fatty acid metabolism prognostic signature (FAMS). The TCGA RNA-seq data were used as the discovery cohort, and 101 kinds of prediction models were fitted using different combinations of the 10 algorithms. Furthermore, we used two RNA-seq datasets (mRNAseq_693 and mRNAseq_325) from the CGGA database as the validation cohort. To avoid the influence of different technologies and platforms, we included three microarray-based expression datasets (TCGA microarray, GSE16011 and GSE13041) as the external validation cohort. The C-index of each model was calculated across all validation cohorts to evaluate its performance. Notably, the model integrated with Lasso and SuperSC achieved the highest average C-index (0.64), outperforming all the other models across the validation cohorts (Figure S3A). In the LASSO regression, 10 FAMGs (*G0S2*, *LDHA*, *ACOT7*, *ADH1C*, *ADH1A*, *APEX1*, *CBR1*, *NBN*, *CD1D*, *GPX2*) with nonzero Lasso coefficients were selected to fit the final model by SuperSC (Figure 3B, Table S3).

All patients in each dataset were assigned to one of two groups on the basis of the median FAMS. Patients in the high-risk group exhibited significantly shorter OS durations than those in the low FAMS group across the training cohort (Figure 3C) and validation cohort (all *p* < 0.05) (Figure 3D-H). We subsequently assessed the predictive value of the FAMS for disease-specific survival (DSS) and progression-free interval (PFI). KM analysis revealed a consistent trend in the RNA-seq data and microarray data, with high-risk patients having shorter DSS and PFI (Figure S3B-E). In addition, Cox regression analysis of the other four datasets including TCGA RNA-seq, TCGA microarray, CGGA mRNAseq_693 and mRNAseq_325 suggested that the FAMS could be an independent prognostic factor. In conclusion, the FAMS demonstrates substantial clinical predictive value for glioblastoma (Figure S3F).

### FAMS model predicts prognosis and treatment response in independent GBM dataset

**Figure 4:**
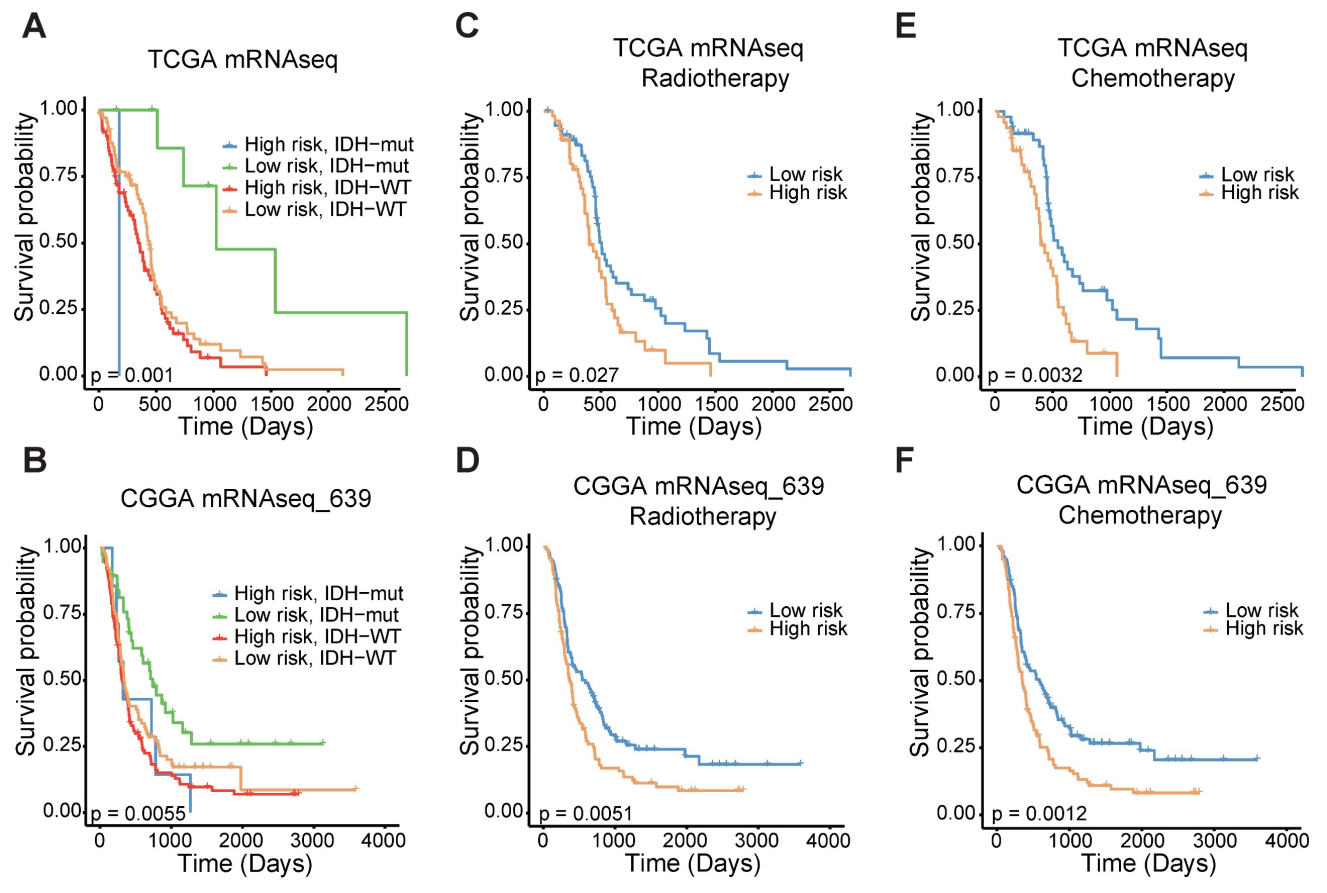
The stratified analysis of other traits and FAMS. **(A)** Kaplan-Meier overall survival analysis among TCGA GBM mRNA-seq patients stratified by FAMS combined with IDH status. **(B)** Kaplan-Meier overall survival analysis among CGGA mRNAseq_639 patients stratified by FAMS combined with IDH status. **(C)** Kaplan-Meier analysis of overall survival among TCGA GBM mRNA-seq patients with radiotherapy. **(D)** Kaplan-Meier analysis of overall survival among CGGA mRNAseq_639 patients with radiotherapy. **(E)** Kaplan-Meier analysis of overall survival among TCGA GBM mRNA-seq patients with chemotherapy. **(F)** Kaplan-Meier analysis of overall survival among CGGA mRNAseq_639 patients with chemotherapy.

To confirm the prognostic significance of the FAMS, we analyzed the discrepancies in OS stratified by IDH mutation status and treatment type. Considering the IDH status, the patients with high FAMS had the worst outcome among the IDH-wild-type patients. Patients with low FAMS and IDH mutation had better survival (Figure 4A). In the CGGA mRNAseq_693 dataset, we observed similar results (Figure 4B). In addition, other datasets also consistently verified that wild-type IDH and high FAMS are associated with worse survival outcomes (Figure S4A-C). When focusing on treatment, high FAMS was also associated with poor outcomes in patients treated with temozolomide (TMZ) chemotherapy and radiotherapy (Figure 4C, E), suggesting that the risk score could serve as a predictor for treatment response. The result was validated in CGGA mRNAseq_693 dataset (Figure 4D, F). In the other three datasets with treatment information, the same result was found in patients treated with radiotherapy (Figure S4D-F), and there was no considerable difference between high- and low-FAMS patients who received chemotherapy (Figure S4G, H). In summary, these findings suggest that the FAMS can serve as a reliable predictor of both prognosis and response to radiotherapy in patients with GBM.

### Functional characterization between the high- and low-FMAS groups

**Figure 5:**
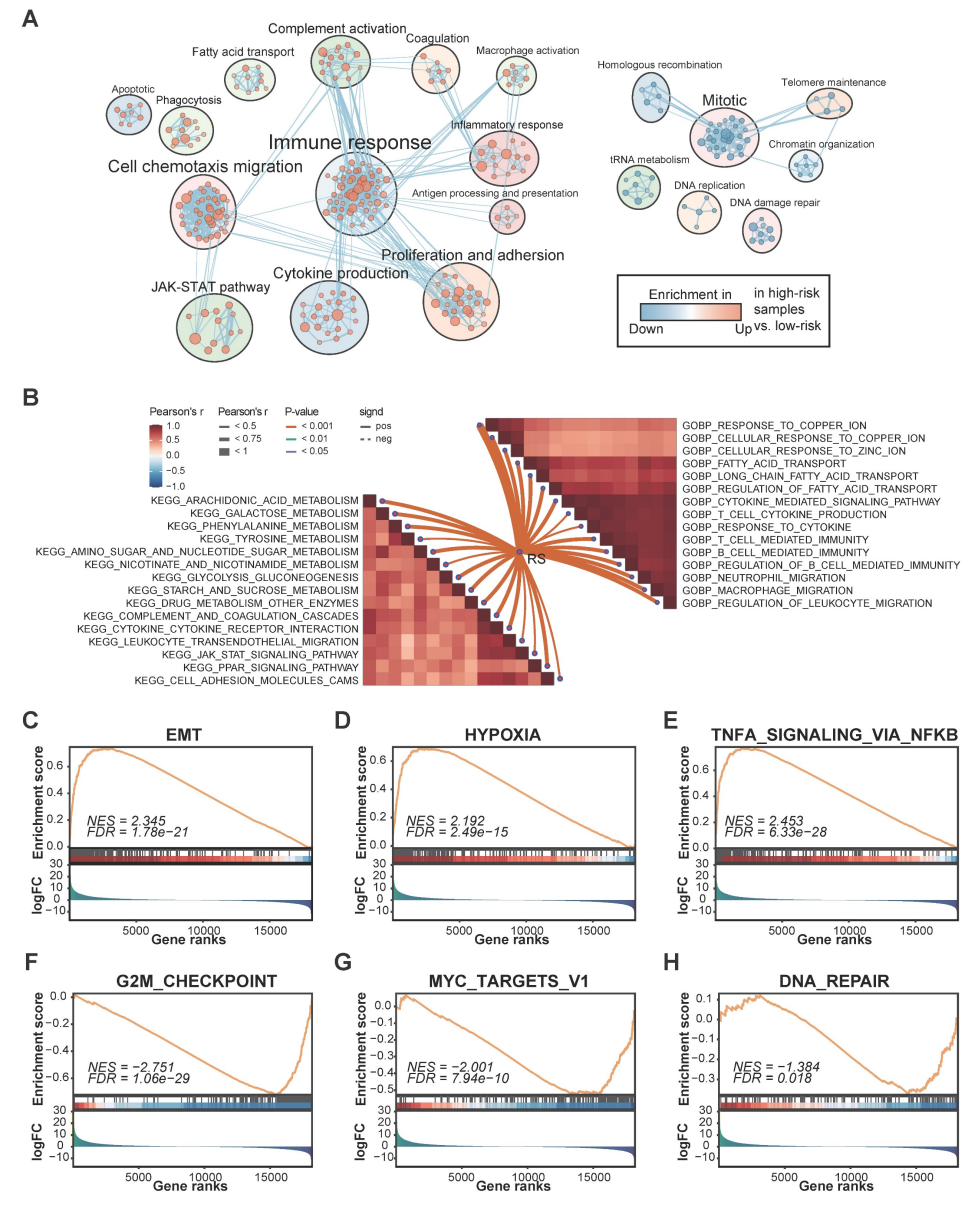
The biological function associated with FAMS. **(A)** Enrichment map showed biological function in high and low FAMS group through GSEA analysis. Each node represents enriched gene sets with *p* < 0.05. Node size corresponds to the number of genes within gene set. Edge thickness corresponds to the number of shared genes between gene sets. **(B)** Butterfly plot showed the correlation between FAMS and other pathway scores based on GSVA of GO and KEGG terms. **(C-H)** GSEA of cancer hallmark associated with high and low FAMS.

Given the excellent performance in predicting survival, we next aimed to explore the underlying mechanisms related to FAMS. Utilizing various functional annotation gene sets, we used GSEA to comprehensively screen and characterize the biological functions involved in the two FAMS groups. Through enrichment analysis, we found that multiple pathways related to immune responses, cell proliferation, fatty acid transport, and cell chemotaxis migration were enriched in the high FAMS score group, whereas the main biological functions involved in the low FAMS score group of patients were Mitotic, DNA damage repair, and homologous recombination (Figure 5A). Moreover, a correlation analysis of the GO terms revealed a positive correlation between FAMS and immune functions, including neutrophil and macrophage migration and T cell and B cell mediated immunity, and the cellular response to ions (zinc and copper) had a similar positive correlation with FAMS (Figure 5B). KEGG analysis showed that the FMAS was strongly associated with pathways such as galactose metabolism, arachidonic acid metabolism, and phenylalanine metabolism (Figure 5B). According to the results of the GSEA of cancer hallmarks, the high FAMS group was enriched in epithelial-to-mesenchymal transition (EMT), hypoxia, and TNFA signaling via NF-κB (Figure 5C-E), and the low FAMS group was enriched in the G2M checkpoint, MYC targets V1, and DNA repair (Figure 5F-H). These results were consistent with the OS analysis results mentioned above. In summary, the results of this analysis suggest that high FMAS is linked to immune responses and may be associated with a superior response to immunotherapy.

### Association of FAMS with the immune program and environment

**Figure 6:**
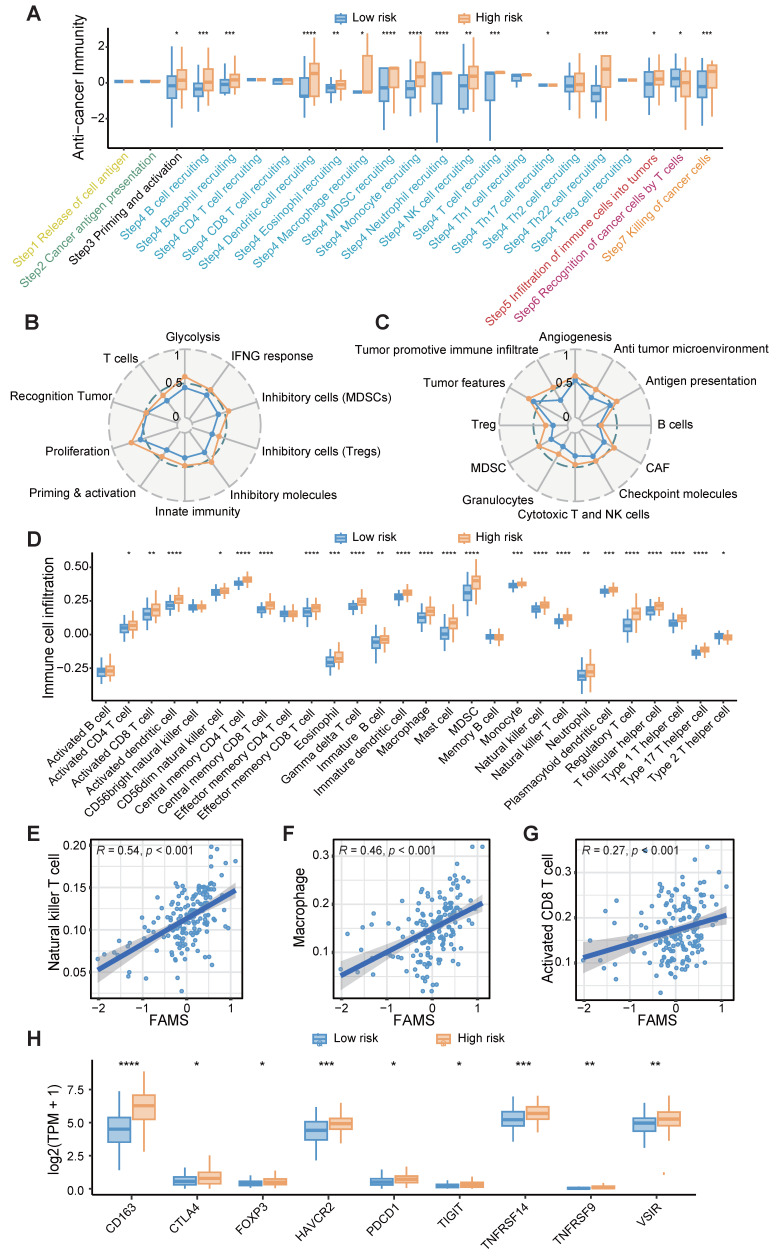
The immune characteristic of FAMS. **(A)** Box plot showed the differences in the cancer immunity cycle between high and low FAMS group. **(B-C)** The radar plot showed the correlation between FAMS and TME signatures developed by Kobayashi and Bagaev. **(D)** Box plot showed the differences in the tumor environment immune cell proportion between high and low FAMS group. **(E-G)** The scatter plot showed the correlation between FAMS and Natural killer T cell, Macrophage, Activated CD8 T cell proportion. **(H)** The expression of immune-related genes in high and low FAMS group. For (A, D, H), p values were calculated by two-sided Wilcoxon rank sum test, the asterisk represents the significance of the difference, *p < 0.05; **p < 0.01; ***p < 0.001.

Owing to the enrichment of immune response-related functions in the high FAMS group, we analyzed the representative steps involved in the cancer immune cycle, including the release of antigens, cancer antigen presentation, priming and activation, immune cell recruitment and infiltration, recognition of cancer cells and killing of cancer cells, and found that immune cell recruitment and cancer cell killing may be more pronounced in the high FAMS group in the TCGA RNA-seq dataset (Figure 6A). Furthermore, we constructed two other immunograms and TME signatures from published literature 46, 47. A radar plot revealed that immune- and TME-related signatures were upregulated in the high FAMS group (Figure 6B-C).

Next, we quantified immune cell infiltration in the TCGA-RNA dataset and explored the relationship between FAMS and immune infiltration. The results showed that compared with the low FAMS group, the high FAMS group had greater proportions of CD8+ T cell, natural killer T cell, and macrophages (Figure 6D). Furthermore, FAMS was positively correlated with the proportions of CD8+ T cell (*R* = 0.27; *p* < 0.001), natural killer T cell (*R* = 0.46; *p* < 0.001), Macrophage (*R* = 0.54; *p* < 0.01) (Figure 6E-G). Additionally, immunosuppressive markers, such as *FOXP3*, *CTLA4*, *CD163*, and *PDCD1,* were more highly expressed in the high-FAMS group (Figure 6H).

### Genomic comparison between the high- and low-FMAS groups

**Figure 7:**
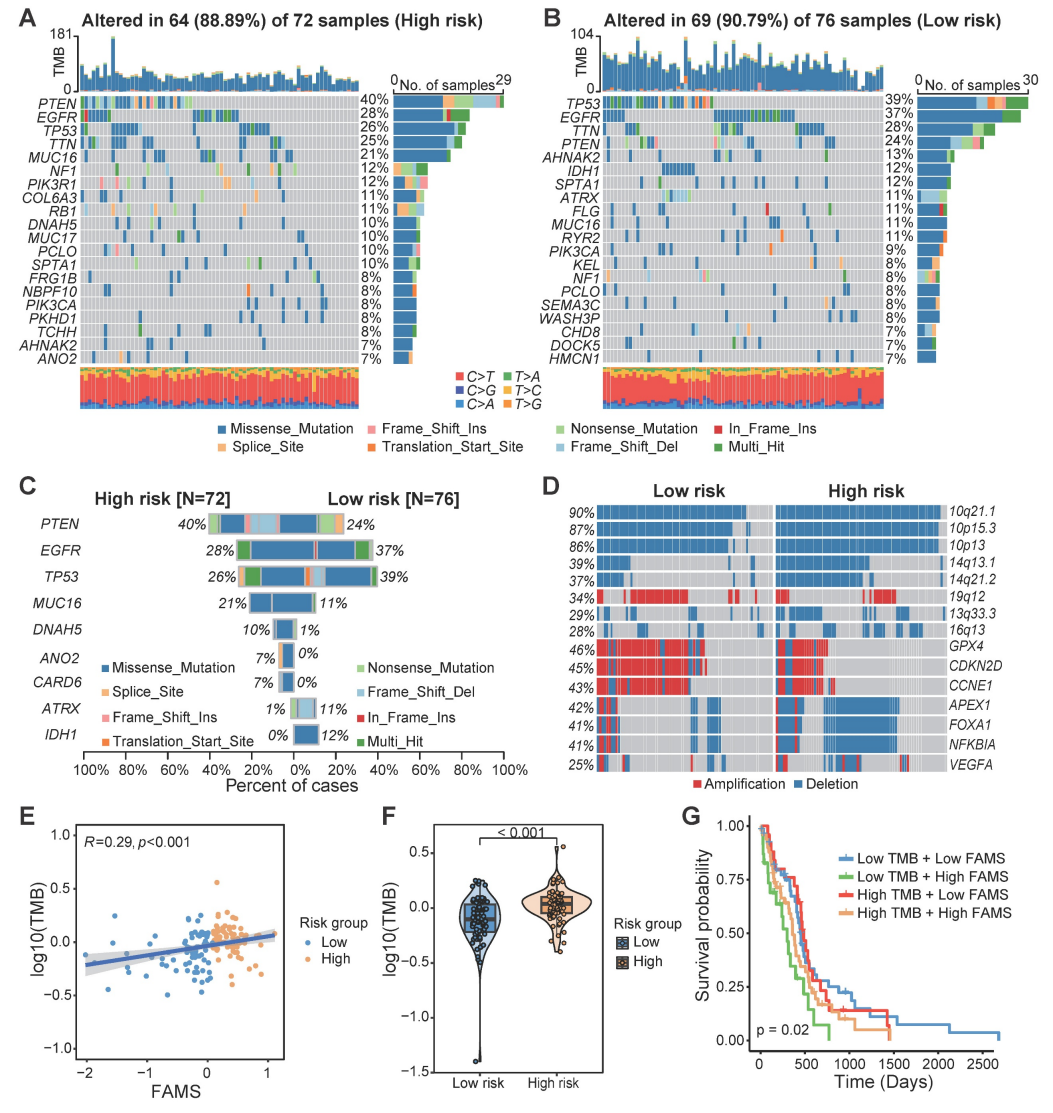
The multiomics alteration characteristics of FAMS. **(A, B)** The gene mutation landscape in high and low FAMS group. Each column represents an individual patient, the top panel showed the tumor mutation burden (TMB), middle panel showed the genomic alternation and the type of genomic aberrations are categorized as follows by their colors: Missense mutation, Frame Shift insertion, Nonsense Mutation, In Frame Insertion, Splice Site mutation, Translation Start Site mutation, Frame Shift Deletion. The bottom panel showed the transition and transversions of SNPs. **(C)** The bar plot showed the genes with significant differences on mutation frequency between high and low FAMS group. **(D)** The CNV profile of high and low FAMS group. **(E)** The scatter plot showed correlation between FAMS and tumor mutation burden. **(F)** Violin plot showed the difference of tumor mutation burden between high and low FAMS group. P values were calculated by two-sided Wilcoxon rank sum test. **(G)** Kaplan-Meier analysis of overall survival combined FAMS and tumor mutation burden.

We compared the somatic mutation profiles of patients in the high-FAMS group and low-FAMS group, and the gene with the highest mutation frequency in the high-FAMS group was *PTEN*, whereas that in the low-FAMS group was *TP53* (Figure 7A, B). Through a chi-square test, we identified several genes with significantly different mutation frequencies between the two patient groups, including *PTEN*, *EGFR*, *TP53*, *ATRX*, and *IDH1* (Figure 7C). Copy number variation analysis revealed that several genes and chromosome segments differed significantly in frequency between the two risk groups (chi-square test, *p < 0.05*). For example, 14q13.1 and 14q21.2 were highly frequently deleted in the high-FAMS group, whereas 19q12 was highly amplified in the low-risk group (Figure 7D). Moreover, patients in the low-risk group exhibited increased amplification of some cell cycle-related genes, such as *CCNE1*, *GPX4*, and *CDKN2D*, and the deletion of the genes *APEX1*, *FOXA1*, *NFKBIA*, and *VEGFA* was frequently observed among patients in the low-FAMS group (Figure 7D). In addition, compared with the low FAMS group, the high FAMS group had a notably greater tumor mutation burden (*p < 0.001*; Figure 7E), and a positive correlation between the TMB and FAMS was observed (Figure 7F). Further categorization of patients by both FAMS score and TMB revealed that the worst prognosis is associated with low TMB and high FAMS scores (Figure 7G). These results emphasize the importance of evaluating both the FAMS and the TMB as critical prognostic factors for predicting patient outcomes.

## Discussion

Tumors in the brain may progress more rapidly because of their specific physiological location and environment 55. Glioblastoma is the most aggressive type of primary brain tumor; and is currently incurable and has a dismal prognosis 56. It is shaped by high heterogeneity of genetic drivers, metabolic programs, and tumor microenvironments 24, 57, 58. Fatty acids, a class of small carbon-rich molecules, play various roles in tumorigenesis and tumor progression. Oxidative phosphorylation (OXPHOS) is crucial for the growth and proliferation of tumor cells 59. However, in the context of hypoxia, nutrient deprivation, and other challenging conditions, fatty acid oxidation (FAO) plays a central role in providing energy to cancer cells. This process significantly affects tumor progression and metastasis 60, 61. Considering the unique environment of the brain, targeting the metabolism of fatty acids could be an effective approach for the treatment of glioblastoma 62, 63. In this study, we extensively characterized the fatty acid metabolism genes in glioblastoma and established a robust prognostic signature that has the potential to aid precision medicine and provide valuable insights into clinical and immunological outcomes (Figure S5). Additionally, these findings can facilitate more detailed investigations on fatty acid metabolism in the future.

In this study, we analyzed the copy number variation, expression levels, and related functional background of FAMGs. The FAMGs with high copy number deletions are related mainly to fatty acid biosynthesis; and elongation, and the genes with high amplification are involved in arachidonic acid metabolism and the adipocytokine signaling pathway. Using the gene expression profiles, patients from the TCGA GBM RNA-seq dataset were stratified into two distinct molecular groups. Cluster 1 expressed higher levels of immune checkpoint genes and had greater immune cell abundance, whereas Cluster 2 was associated with a more favorable prognosis. DEGs between the two groups were identified. GO and KEGG analyses revealed that the genes whose expression was high in Cluster 1 might participate in biological processes and pathways related to the immune response. These findings may indicate that the expression patterns of FAMGs are potentially related to the tumor microenvironment of GBM, which may result in different survival outcomes and immune response states.

On the basis of the differential expression of FAMGs and their association with prognosis, we constructed a prognostic signature, referred to as FAMS, to predict patient survival and prognosis. All patients were divided into high- and low-FAMS groups on the basis of the FAMS values, which demonstrated disparate survival trends and biological characteristics. The patients in the high FAMS group exhibited consistently shorter OS, DSS, and PFI across multiple datasets from different data platforms. In addition, fatty acid metabolism has been reported to be associated with resistance to radiotherapy in tumors 64-67, and the FAMS in our study could distinguish the patients who exhibit a greater response to radiotherapy. The patients with low FAMS showed prolonged survival, which may indicate that they had a superior response to radiotherapy. We also observed an association between FAMS and chemotherapy response, but the association was not consistently significant. Each of these genes plays a unique role in tumorigenesis and tumor progression. For example, *G0S2* is a gene involved in extrinsic apoptotic signaling pathway, and is considered a key regulator of energy homeostasis, controlling both fatty acid availability and fatty acid oxidation 68, 69. *ACOT7* is an Acyl-CoA thioesterase isoform that is involved primarily in the hydrolysis of arachidonoyl-CoA. It can provide the arachidonic acid required for the synthesis of prostaglandins 70. While the arachidonic acid can serve as a pro-inflammatory precursor, it is essential for the cell cycle, cell proliferation, and glucose metabolism 71, 72. Overall, additional research is necessary to clarify the specific molecular mechanisms and potential roles of these genes.

A number of studies have indicated that immunotherapy may benefit patients with glioblastoma; nevertheless, owing to the lack of understanding of the tumor environment and immune processes, a considerable proportion of patients derive only minimal benefit from immunotherapy 73, 74. We evaluated the anticancer immune process through a seven-step Cancer-Immunity Cycle, including the release of cancer cell antigens, cancer antigen presentation, priming and activation, trafficking of immune cells to tumors, infiltration of immune cells into tumors, recognition of cancer cells by T cells, and killing of cancer cells 45. Patients in the high FAMS group had high functional scores related to immune cell recruitment. FAMS was positively correlated with the immune cell proportions of diverse immune cell types, including macrophage, activated CD8+ T cell, and natural killer T cell. This may indicate a potential immune response in patients with high FAMS.

In summary, we developed a prognostic signature derived from FAMGs. This model can serve as an independent prognostic factor to predict the outcomes of GBM patients. Nonetheless, a key limitation of our study is the absence of validation in a prospective cohort; and the lack of functional validation for the genes incorporated in FAMS. In addition, clinical and molecular information in the public data was limited, so there may be some potential associations with FAMS that were not observed in this study.

## Conclusions

Within the confines of this study, we systematically explored the multiomics landscape of fatty acid metabolism genes (FAMGs) in glioblastoma and meticulously devised a robust prognostic signature by integrating FAMGs. In addition, this research elucidates the potential relationships between prognostic models and mutation patterns, the tumor microenvironment, and biological functions. Consequently, this signature has the potential to serve as a robust and promising tool to improve personalized risk stratification and therapeutic implications for glioblastoma patients.

## Supplementary Material

Supplementary figures and tables.
