# Supplementary material for: Robust machine-learning based prognostic index using fatty acid metabolism genes predicts prognosis and therapy responses in glioblastoma
Source: J Cancer. 2025 Aug 22;16(13):3859–73. doi: 10.7150/jca.117209 (PMC12490975; doi:10.7150/jca.117209)
Supplement: Supplementary file 1 — Supplementary figures and tables. [file jcav16p3859s1.zip › Supplemental information.pdf]

Supplementary Figures

Figure S1

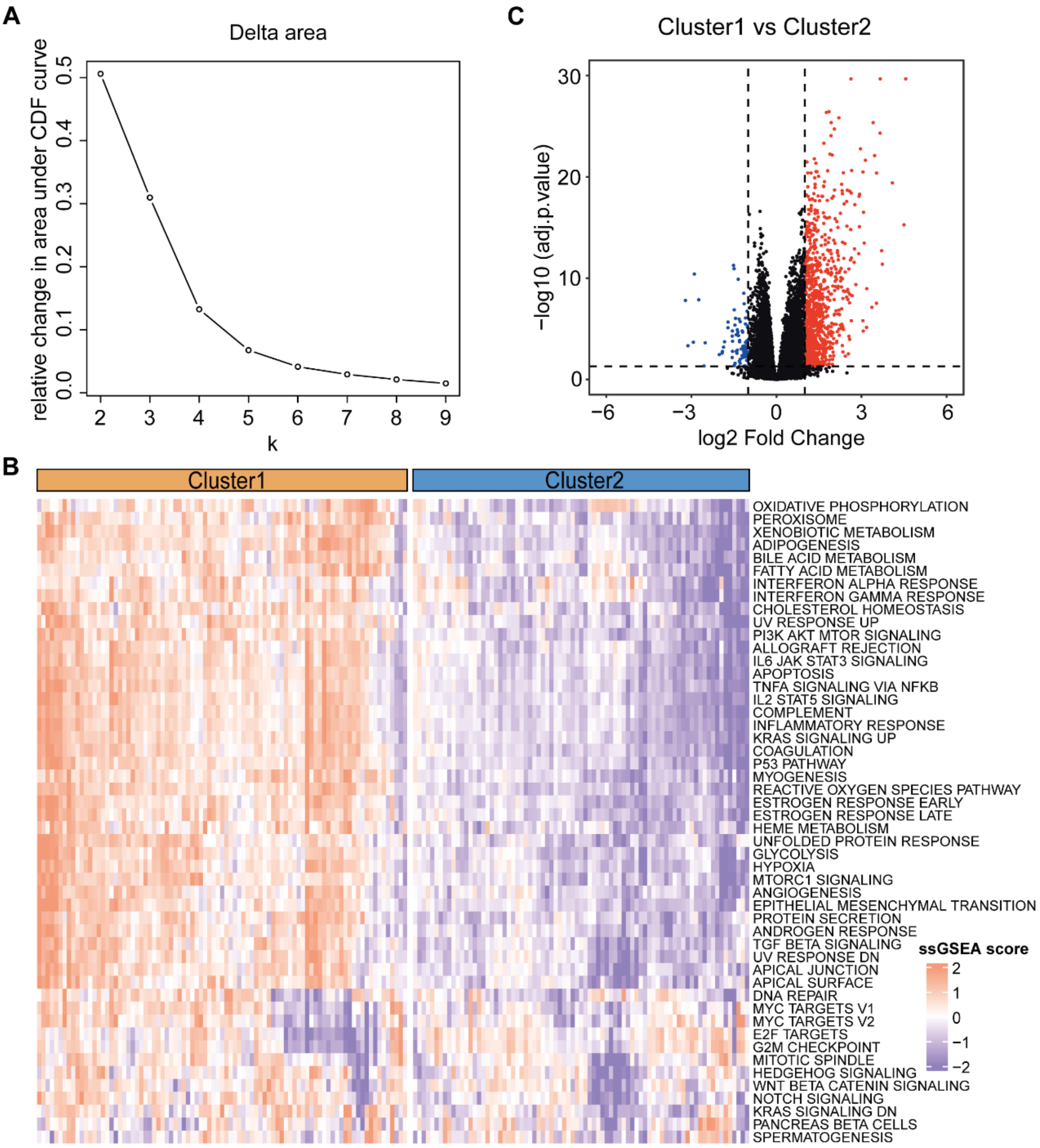

Figure S1: Consensus clustering of FAMGs.

(A) The line plot of relative change in area under CDF curve for each k. **(B)** The heatmap showed the hallmark function difference between the FAMGs cluster 1 and cluster 2. **(C)** The volcano plot showed the genes differently expressed between cluster 1 and cluster 2.

Figure S2

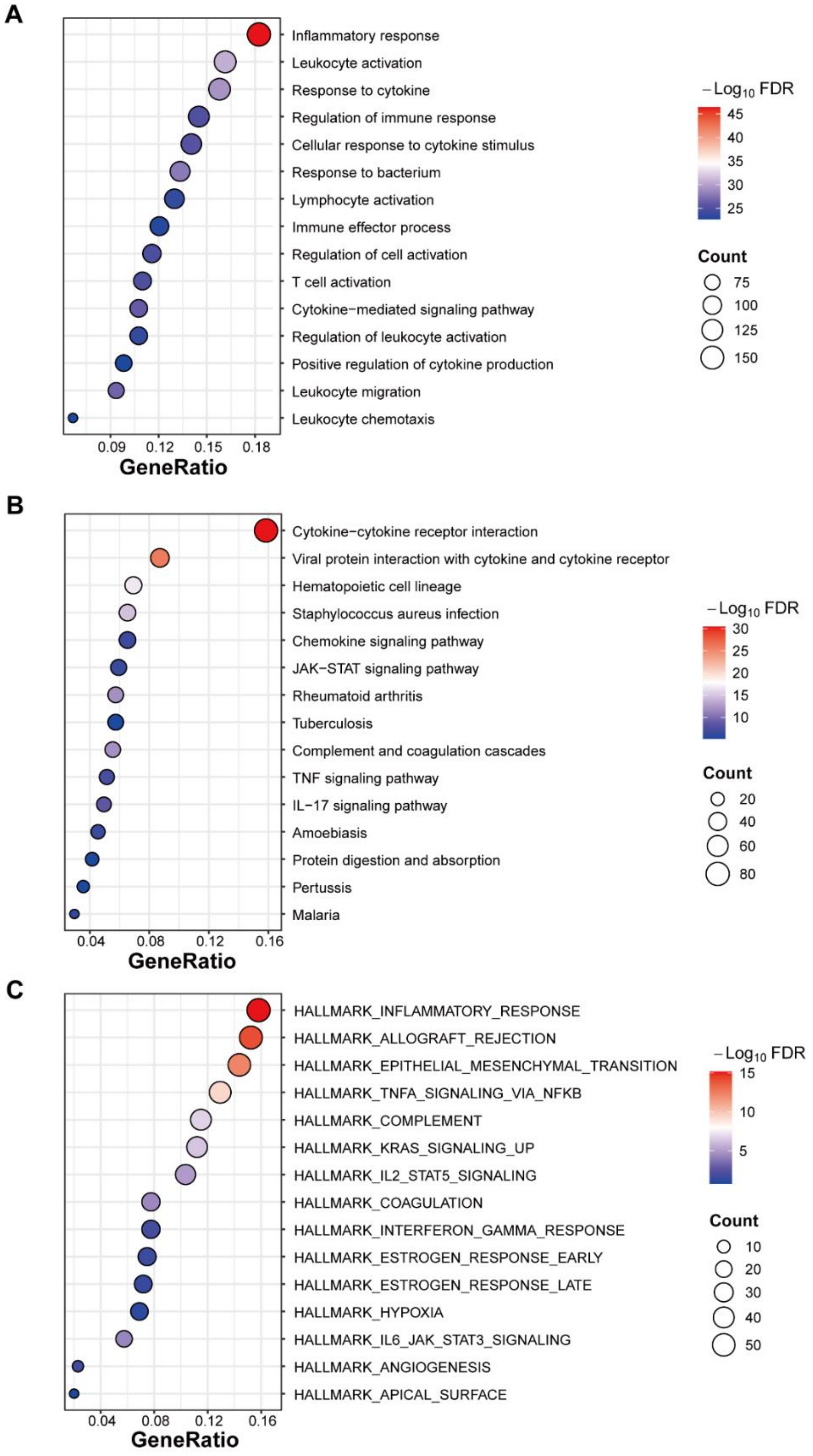

Figure S2: The function enrichment analysis of upregulated genes in cluster 1.

(A) the enrichment dot plot showed the enriched function of GO terms. **(B)** the enrichment dot plot showed the enriched function of KEGG pathways. **(C)** the enrichment dot plot showed the enriched function of cancer hallmark.

Figure S3

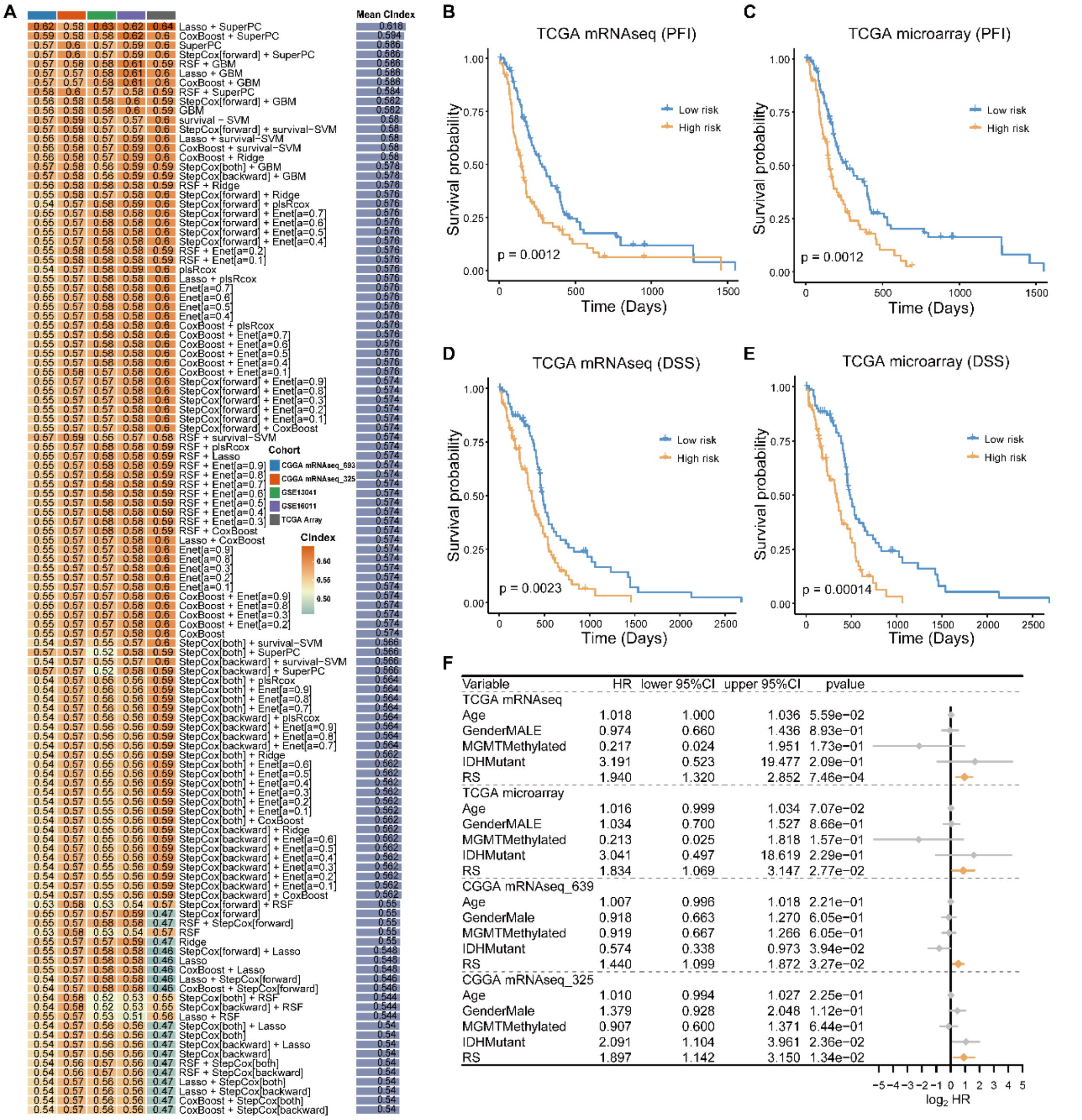

Figure S3: Calculation of FAMS using of machine learning algorithm combination. **(A)** Total of 101 kinds of prediction models constructed by different algorithm combinations and further calculated the C-index of each model in all validation datasets. **(B-C)** Kaplan–Meier curves of progression free interval (PFI) in TCGA GBM mRNA-seq and microarray dataset. **(D-E)** Kaplan–Meier curves of Disease Specific Survival (DSS) in TCGA GBM mRNA-seq and microarray dataset. **(F)** Multivariate Cox regression analysis of FAMS in TCGA mRNA-seq, TCGA microarray, CGGA mRNAseq_639, and CGGA mRNAseq_325.

Figure S4

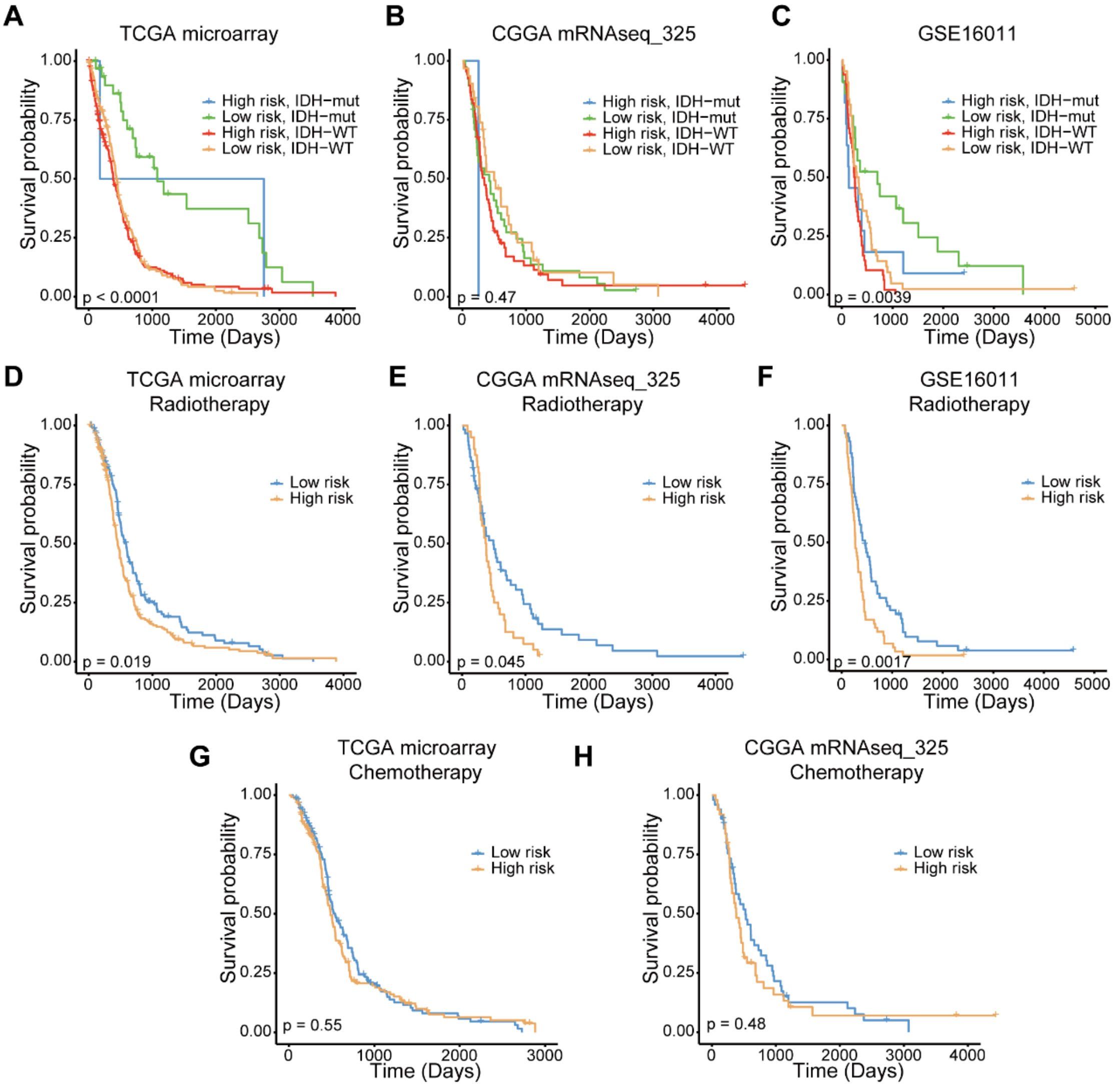

Figure S4: The stratified analysis of IDH mutation and treatment with FAMS.

(A-C) Kaplan–Meier overall survival analysis of FAMS combined with IDH status in TCGA microarray, CGGA mRNAseq_325, and GSE16011 dataset. **(D-F)** Kaplan–Meier overall survival analysis of FAMS among patients with radiotherapy in TCGA microarray, CGGA mRNAseq_325, and GSE16011 dataset. **(G, H)** Kaplan–Meier overall survival analysis of FAMS among patients with chemotherapy in TCGA microarray, CGGA mRNAseq_325 dataset.

Figure S5

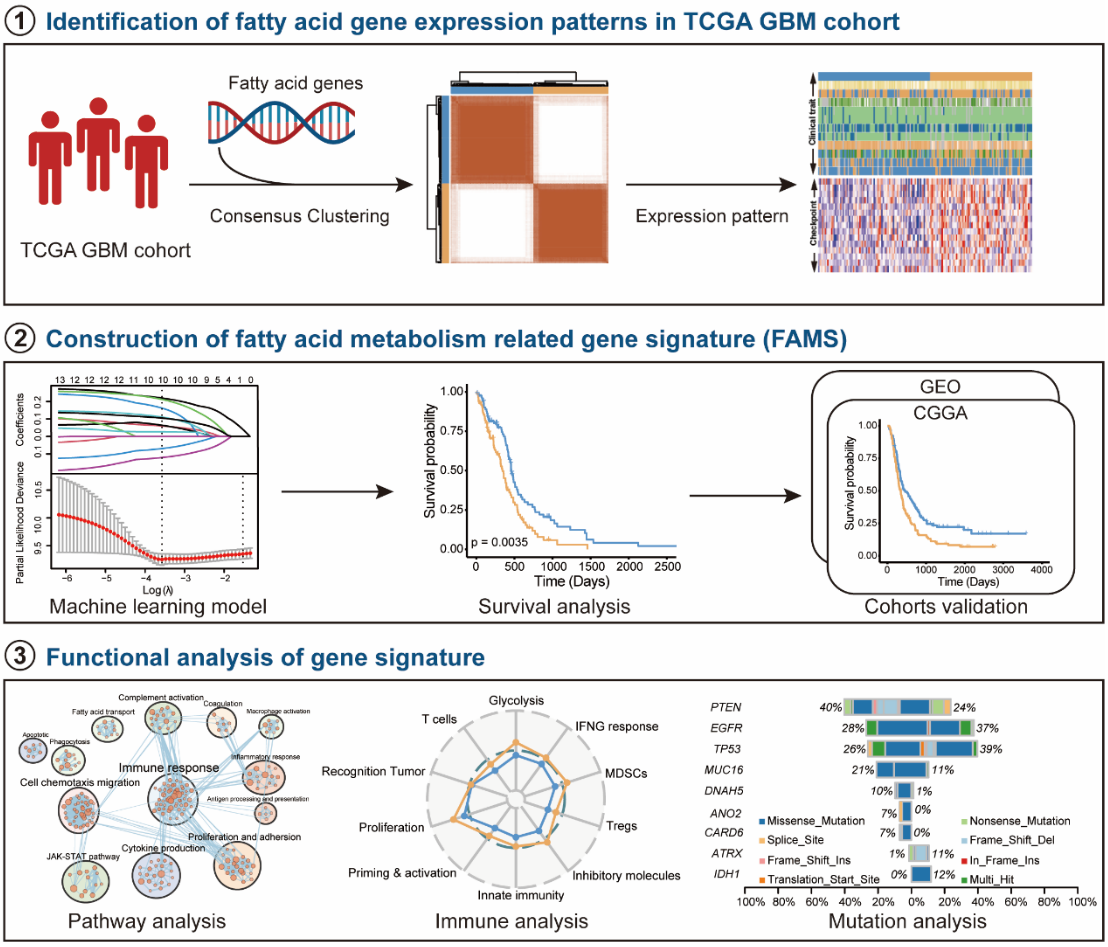

Figure S5: The workflow of the present study.

(1) Identification fatty acid metabolism related genes expression pattern in TCGA GBM cohort. (2) Constructing fatty acid metabolism-related prognostic signature (FAMS) based on machine learning model. (3) Functional characterization of FAMS through pathway analysis, immune infiltration analysis and mutation analysis.
