# Supplementary material for: Robust machine-learning based prognostic index using fatty acid metabolism genes predicts prognosis and therapy responses in glioblastoma
Source: J Cancer. 2025 Aug 22;16(13):3859–73. doi: 10.7150/jca.117209 (PMC12490975; doi:10.7150/jca.117209)
Supplement: Supplementary file 1 — Supplementary figures and tables. [file jcav16p3859s1.zip › Supplemental TableS1.docx]

**Table S1. The gene list of fatty acid metabolism related genes**

| **Source** | **SYMBOL** |
| --- | --- |
| HALLMARK_FATTY_ACID_METABOLISM | AADAT |
| HALLMARK_FATTY_ACID_METABOLISM | ACAA1 |
| HALLMARK_FATTY_ACID_METABOLISM | ACAA2 |
| HALLMARK_FATTY_ACID_METABOLISM | ACADL |
| HALLMARK_FATTY_ACID_METABOLISM | ACADM |
| HALLMARK_FATTY_ACID_METABOLISM | ACADS |
| HALLMARK_FATTY_ACID_METABOLISM | ACADVL |
| HALLMARK_FATTY_ACID_METABOLISM | ACAT2 |
| HALLMARK_FATTY_ACID_METABOLISM | ACO2 |
| HALLMARK_FATTY_ACID_METABOLISM | ACOT2 |
| HALLMARK_FATTY_ACID_METABOLISM | ACOT8 |
| HALLMARK_FATTY_ACID_METABOLISM | ACOX1 |
| HALLMARK_FATTY_ACID_METABOLISM | ACSL1 |
| HALLMARK_FATTY_ACID_METABOLISM | ACSL4 |
| HALLMARK_FATTY_ACID_METABOLISM | ACSL5 |
| HALLMARK_FATTY_ACID_METABOLISM | ACSM3 |
| HALLMARK_FATTY_ACID_METABOLISM | ACSS1 |
| HALLMARK_FATTY_ACID_METABOLISM | ADH1C |
| HALLMARK_FATTY_ACID_METABOLISM | ADH7 |
| HALLMARK_FATTY_ACID_METABOLISM | ADIPOR2 |
| HALLMARK_FATTY_ACID_METABOLISM | ADSL |
| HALLMARK_FATTY_ACID_METABOLISM | ALAD |
| HALLMARK_FATTY_ACID_METABOLISM | ALDH1A1 |
| HALLMARK_FATTY_ACID_METABOLISM | ALDH3A1 |
| HALLMARK_FATTY_ACID_METABOLISM | ALDH3A2 |
| HALLMARK_FATTY_ACID_METABOLISM | ALDH9A1 |
| HALLMARK_FATTY_ACID_METABOLISM | ALDOA |
| HALLMARK_FATTY_ACID_METABOLISM | AOC3 |
| HALLMARK_FATTY_ACID_METABOLISM | APEX1 |
| HALLMARK_FATTY_ACID_METABOLISM | AQP7 |
| HALLMARK_FATTY_ACID_METABOLISM | AUH |
| HALLMARK_FATTY_ACID_METABOLISM | BCKDHB |
| HALLMARK_FATTY_ACID_METABOLISM | BLVRA |
| HALLMARK_FATTY_ACID_METABOLISM | BMPR1B |
| HALLMARK_FATTY_ACID_METABOLISM | BPHL |
| HALLMARK_FATTY_ACID_METABOLISM | CA2 |
| HALLMARK_FATTY_ACID_METABOLISM | CA4 |
| HALLMARK_FATTY_ACID_METABOLISM | CA6 |
| HALLMARK_FATTY_ACID_METABOLISM | CBR1 |
| HALLMARK_FATTY_ACID_METABOLISM | CBR3 |
| HALLMARK_FATTY_ACID_METABOLISM | MIX23 |
| HALLMARK_FATTY_ACID_METABOLISM | CD1D |
| HALLMARK_FATTY_ACID_METABOLISM | CD36 |
| HALLMARK_FATTY_ACID_METABOLISM | CEL |
| HALLMARK_FATTY_ACID_METABOLISM | CIDEA |
| HALLMARK_FATTY_ACID_METABOLISM | CPOX |
| HALLMARK_FATTY_ACID_METABOLISM | CPT1A |
| HALLMARK_FATTY_ACID_METABOLISM | CPT2 |
| HALLMARK_FATTY_ACID_METABOLISM | CRAT |
| HALLMARK_FATTY_ACID_METABOLISM | CRYZ |
| HALLMARK_FATTY_ACID_METABOLISM | CYP1A1 |
| HALLMARK_FATTY_ACID_METABOLISM | CYP4A11 |
| HALLMARK_FATTY_ACID_METABOLISM | CYP4A22 |
| HALLMARK_FATTY_ACID_METABOLISM | D2HGDH |
| HALLMARK_FATTY_ACID_METABOLISM | DECR1 |
| HALLMARK_FATTY_ACID_METABOLISM | DHCR24 |
| HALLMARK_FATTY_ACID_METABOLISM | DLD |
| HALLMARK_FATTY_ACID_METABOLISM | DLST |
| HALLMARK_FATTY_ACID_METABOLISM | ECH1 |
| HALLMARK_FATTY_ACID_METABOLISM | ECHS1 |
| HALLMARK_FATTY_ACID_METABOLISM | ECI1 |
| HALLMARK_FATTY_ACID_METABOLISM | ECI2 |
| HALLMARK_FATTY_ACID_METABOLISM | EHHADH |
| HALLMARK_FATTY_ACID_METABOLISM | ELOVL5 |
| HALLMARK_FATTY_ACID_METABOLISM | ENO2 |
| HALLMARK_FATTY_ACID_METABOLISM | ENO3 |
| HALLMARK_FATTY_ACID_METABOLISM | EPHX1 |
| HALLMARK_FATTY_ACID_METABOLISM | ERP29 |
| HALLMARK_FATTY_ACID_METABOLISM | ETFDH |
| HALLMARK_FATTY_ACID_METABOLISM | FABP1 |
| HALLMARK_FATTY_ACID_METABOLISM | FABP2 |
| HALLMARK_FATTY_ACID_METABOLISM | FASN |
| HALLMARK_FATTY_ACID_METABOLISM | FH |
| HALLMARK_FATTY_ACID_METABOLISM | FMO1 |
| HALLMARK_FATTY_ACID_METABOLISM | G0S2 |
| HALLMARK_FATTY_ACID_METABOLISM | GABARAPL1 |
| HALLMARK_FATTY_ACID_METABOLISM | GAD2 |
| HALLMARK_FATTY_ACID_METABOLISM | GAPDHS |
| HALLMARK_FATTY_ACID_METABOLISM | GCDH |
| HALLMARK_FATTY_ACID_METABOLISM | GLUL |
| HALLMARK_FATTY_ACID_METABOLISM | GPD1 |
| HALLMARK_FATTY_ACID_METABOLISM | GPD2 |
| HALLMARK_FATTY_ACID_METABOLISM | GRHPR |
| HALLMARK_FATTY_ACID_METABOLISM | GSTZ1 |
| HALLMARK_FATTY_ACID_METABOLISM | H2AZ1 |
| HALLMARK_FATTY_ACID_METABOLISM | HADH |
| HALLMARK_FATTY_ACID_METABOLISM | HADHB |
| HALLMARK_FATTY_ACID_METABOLISM | HAO2 |
| HALLMARK_FATTY_ACID_METABOLISM | HCCS |
| HALLMARK_FATTY_ACID_METABOLISM | HIBCH |
| HALLMARK_FATTY_ACID_METABOLISM | HMGCL |
| HALLMARK_FATTY_ACID_METABOLISM | HMGCS1 |
| HALLMARK_FATTY_ACID_METABOLISM | HMGCS2 |
| HALLMARK_FATTY_ACID_METABOLISM | HPGD |
| HALLMARK_FATTY_ACID_METABOLISM | HSD17B10 |
| HALLMARK_FATTY_ACID_METABOLISM | HSD17B11 |
| HALLMARK_FATTY_ACID_METABOLISM | HSD17B4 |
| HALLMARK_FATTY_ACID_METABOLISM | HSD17B7 |
| HALLMARK_FATTY_ACID_METABOLISM | HSDL2 |
| HALLMARK_FATTY_ACID_METABOLISM | HSP90AA1 |
| HALLMARK_FATTY_ACID_METABOLISM | HSPH1 |
| HALLMARK_FATTY_ACID_METABOLISM | IDH1 |
| HALLMARK_FATTY_ACID_METABOLISM | IDH3B |
| HALLMARK_FATTY_ACID_METABOLISM | IDH3G |
| HALLMARK_FATTY_ACID_METABOLISM | IDI1 |
| HALLMARK_FATTY_ACID_METABOLISM | IL4I1 |
| HALLMARK_FATTY_ACID_METABOLISM | INMT |
| HALLMARK_FATTY_ACID_METABOLISM | LDHA |
| HALLMARK_FATTY_ACID_METABOLISM | LGALS1 |
| HALLMARK_FATTY_ACID_METABOLISM | LTC4S |
| HALLMARK_FATTY_ACID_METABOLISM | MAOA |
| HALLMARK_FATTY_ACID_METABOLISM | MCEE |
| HALLMARK_FATTY_ACID_METABOLISM | MDH1 |
| HALLMARK_FATTY_ACID_METABOLISM | MDH2 |
| HALLMARK_FATTY_ACID_METABOLISM | ME1 |
| HALLMARK_FATTY_ACID_METABOLISM | METAP1 |
| HALLMARK_FATTY_ACID_METABOLISM | MGLL |
| HALLMARK_FATTY_ACID_METABOLISM | MIF |
| HALLMARK_FATTY_ACID_METABOLISM | MLYCD |
| HALLMARK_FATTY_ACID_METABOLISM | NBN |
| HALLMARK_FATTY_ACID_METABOLISM | NCAPH2 |
| HALLMARK_FATTY_ACID_METABOLISM | NSDHL |
| HALLMARK_FATTY_ACID_METABOLISM | NTHL1 |
| HALLMARK_FATTY_ACID_METABOLISM | ODC1 |
| HALLMARK_FATTY_ACID_METABOLISM | OSTC |
| HALLMARK_FATTY_ACID_METABOLISM | PCBD1 |
| HALLMARK_FATTY_ACID_METABOLISM | PDHA1 |
| HALLMARK_FATTY_ACID_METABOLISM | PDHB |
| HALLMARK_FATTY_ACID_METABOLISM | PPARA |
| HALLMARK_FATTY_ACID_METABOLISM | PRDX6 |
| HALLMARK_FATTY_ACID_METABOLISM | PSME1 |
| HALLMARK_FATTY_ACID_METABOLISM | PTPRG |
| HALLMARK_FATTY_ACID_METABOLISM | PTS |
| HALLMARK_FATTY_ACID_METABOLISM | RAP1GDS1 |
| HALLMARK_FATTY_ACID_METABOLISM | RDH11 |
| HALLMARK_FATTY_ACID_METABOLISM | RDH16 |
| HALLMARK_FATTY_ACID_METABOLISM | REEP6 |
| HALLMARK_FATTY_ACID_METABOLISM | RETSAT |
| HALLMARK_FATTY_ACID_METABOLISM | S100A10 |
| HALLMARK_FATTY_ACID_METABOLISM | SDHA |
| HALLMARK_FATTY_ACID_METABOLISM | SDHC |
| HALLMARK_FATTY_ACID_METABOLISM | SDHD |
| HALLMARK_FATTY_ACID_METABOLISM | SERINC1 |
| HALLMARK_FATTY_ACID_METABOLISM | KMT5A |
| HALLMARK_FATTY_ACID_METABOLISM | SLC22A5 |
| HALLMARK_FATTY_ACID_METABOLISM | SMS |
| HALLMARK_FATTY_ACID_METABOLISM | SUCLA2 |
| HALLMARK_FATTY_ACID_METABOLISM | SUCLG1 |
| HALLMARK_FATTY_ACID_METABOLISM | SUCLG2 |
| HALLMARK_FATTY_ACID_METABOLISM | TDO2 |
| HALLMARK_FATTY_ACID_METABOLISM | TP53INP2 |
| HALLMARK_FATTY_ACID_METABOLISM | UBE2L6 |
| HALLMARK_FATTY_ACID_METABOLISM | UGDH |
| HALLMARK_FATTY_ACID_METABOLISM | UROD |
| HALLMARK_FATTY_ACID_METABOLISM | UROS |
| HALLMARK_FATTY_ACID_METABOLISM | VNN1 |
| HALLMARK_FATTY_ACID_METABOLISM | XIST |
| HALLMARK_FATTY_ACID_METABOLISM | YWHAH |
| KEGG_FATTY_ACID_METABOLISM | ACAA2 |
| KEGG_FATTY_ACID_METABOLISM | ECI2 |
| KEGG_FATTY_ACID_METABOLISM | ADH1A |
| KEGG_FATTY_ACID_METABOLISM | ADH1B |
| KEGG_FATTY_ACID_METABOLISM | ADH1C |
| KEGG_FATTY_ACID_METABOLISM | CPT1C |
| KEGG_FATTY_ACID_METABOLISM | ADH4 |
| KEGG_FATTY_ACID_METABOLISM | ADH5 |
| KEGG_FATTY_ACID_METABOLISM | ADH6 |
| KEGG_FATTY_ACID_METABOLISM | ADH7 |
| KEGG_FATTY_ACID_METABOLISM | CPT1A |
| KEGG_FATTY_ACID_METABOLISM | CPT1B |
| KEGG_FATTY_ACID_METABOLISM | CPT2 |
| KEGG_FATTY_ACID_METABOLISM | CYP4A11 |
| KEGG_FATTY_ACID_METABOLISM | ECI1 |
| KEGG_FATTY_ACID_METABOLISM | ECHS1 |
| KEGG_FATTY_ACID_METABOLISM | EHHADH |
| KEGG_FATTY_ACID_METABOLISM | ALDH2 |
| KEGG_FATTY_ACID_METABOLISM | ACSL1 |
| KEGG_FATTY_ACID_METABOLISM | ACSL3 |
| KEGG_FATTY_ACID_METABOLISM | ACSL4 |
| KEGG_FATTY_ACID_METABOLISM | ALDH1B1 |
| KEGG_FATTY_ACID_METABOLISM | ALDH9A1 |
| KEGG_FATTY_ACID_METABOLISM | ALDH3A2 |
| KEGG_FATTY_ACID_METABOLISM | ACSL6 |
| KEGG_FATTY_ACID_METABOLISM | GCDH |
| KEGG_FATTY_ACID_METABOLISM | CYP4A22 |
| KEGG_FATTY_ACID_METABOLISM | ACAA1 |
| KEGG_FATTY_ACID_METABOLISM | HADHA |
| KEGG_FATTY_ACID_METABOLISM | HADHB |
| KEGG_FATTY_ACID_METABOLISM | HADH |
| KEGG_FATTY_ACID_METABOLISM | ACADL |
| KEGG_FATTY_ACID_METABOLISM | ACADM |
| KEGG_FATTY_ACID_METABOLISM | ACADS |
| KEGG_FATTY_ACID_METABOLISM | ACADSB |
| KEGG_FATTY_ACID_METABOLISM | ACADVL |
| KEGG_FATTY_ACID_METABOLISM | ACAT1 |
| KEGG_FATTY_ACID_METABOLISM | ACAT2 |
| KEGG_FATTY_ACID_METABOLISM | ALDH7A1 |
| KEGG_FATTY_ACID_METABOLISM | ACOX1 |
| KEGG_FATTY_ACID_METABOLISM | ACSL5 |
| KEGG_FATTY_ACID_METABOLISM | ACOX3 |
| REACTOME_BETA_OXIDATION_OF_VERY_LONG_CHAIN_FATTY_ACIDS | ACAA1 |
| REACTOME_BETA_OXIDATION_OF_VERY_LONG_CHAIN_FATTY_ACIDS | ACOT8 |
| REACTOME_BETA_OXIDATION_OF_VERY_LONG_CHAIN_FATTY_ACIDS | ABCD1 |
| REACTOME_BETA_OXIDATION_OF_VERY_LONG_CHAIN_FATTY_ACIDS | MLYCD |
| REACTOME_BETA_OXIDATION_OF_VERY_LONG_CHAIN_FATTY_ACIDS | EHHADH |
| REACTOME_BETA_OXIDATION_OF_VERY_LONG_CHAIN_FATTY_ACIDS | HSD17B4 |
| REACTOME_BETA_OXIDATION_OF_VERY_LONG_CHAIN_FATTY_ACIDS | ACOX1 |
| REACTOME_BETA_OXIDATION_OF_VERY_LONG_CHAIN_FATTY_ACIDS | ACOT4 |
| REACTOME_BETA_OXIDATION_OF_VERY_LONG_CHAIN_FATTY_ACIDS | ECI2 |
| REACTOME_BETA_OXIDATION_OF_VERY_LONG_CHAIN_FATTY_ACIDS | ACOT6 |
| REACTOME_BETA_OXIDATION_OF_VERY_LONG_CHAIN_FATTY_ACIDS | DECR2 |
| REACTOME_BETA_OXIDATION_OF_VERY_LONG_CHAIN_FATTY_ACIDS | DECR2 |
| REACTOME_FATTY_ACID_METABOLISM | NDUFAB1 |
| REACTOME_FATTY_ACID_METABOLISM | ACSM3 |
| REACTOME_FATTY_ACID_METABOLISM | PON1 |
| REACTOME_FATTY_ACID_METABOLISM | CROT |
| REACTOME_FATTY_ACID_METABOLISM | ELOVL5 |
| REACTOME_FATTY_ACID_METABOLISM | ALOX5 |
| REACTOME_FATTY_ACID_METABOLISM | DPEP1 |
| REACTOME_FATTY_ACID_METABOLISM | TBXAS1 |
| REACTOME_FATTY_ACID_METABOLISM | ACAA1 |
| REACTOME_FATTY_ACID_METABOLISM | ELOVL1 |
| REACTOME_FATTY_ACID_METABOLISM | ACSL4 |
| REACTOME_FATTY_ACID_METABOLISM | ALDH3A2 |
| REACTOME_FATTY_ACID_METABOLISM | ACADVL |
| REACTOME_FATTY_ACID_METABOLISM | PTGS2 |
| REACTOME_FATTY_ACID_METABOLISM | HACD3 |
| REACTOME_FATTY_ACID_METABOLISM | ACACB |
| REACTOME_FATTY_ACID_METABOLISM | HADHA |
| REACTOME_FATTY_ACID_METABOLISM | ACOX3 |
| REACTOME_FATTY_ACID_METABOLISM | PTGS1 |
| REACTOME_FATTY_ACID_METABOLISM | CRAT |
| REACTOME_FATTY_ACID_METABOLISM | ACOT7 |
| REACTOME_FATTY_ACID_METABOLISM | SCD |
| REACTOME_FATTY_ACID_METABOLISM | TECR |
| REACTOME_FATTY_ACID_METABOLISM | GGT5 |
| REACTOME_FATTY_ACID_METABOLISM | GGT1 |
| REACTOME_FATTY_ACID_METABOLISM | MCAT |
| REACTOME_FATTY_ACID_METABOLISM | SLC25A17 |
| REACTOME_FATTY_ACID_METABOLISM | ACOT8 |
| REACTOME_FATTY_ACID_METABOLISM | ABCD1 |
| REACTOME_FATTY_ACID_METABOLISM | MLYCD |
| REACTOME_FATTY_ACID_METABOLISM | ABCC1 |
| REACTOME_FATTY_ACID_METABOLISM | ACSBG1 |
| REACTOME_FATTY_ACID_METABOLISM | DECR1 |
| REACTOME_FATTY_ACID_METABOLISM | PON3 |
| REACTOME_FATTY_ACID_METABOLISM | PON2 |
| REACTOME_FATTY_ACID_METABOLISM | PRKAG2 |
| REACTOME_FATTY_ACID_METABOLISM | PTGR1 |
| REACTOME_FATTY_ACID_METABOLISM | PTGDS |
| REACTOME_FATTY_ACID_METABOLISM | PHYH |
| REACTOME_FATTY_ACID_METABOLISM | ACBD5 |
| REACTOME_FATTY_ACID_METABOLISM | ALOX12 |
| REACTOME_FATTY_ACID_METABOLISM | CPT1A |
| REACTOME_FATTY_ACID_METABOLISM | PTGES3 |
| REACTOME_FATTY_ACID_METABOLISM | LTA4H |
| REACTOME_FATTY_ACID_METABOLISM | ACAD10 |
| REACTOME_FATTY_ACID_METABOLISM | PPARD |
| REACTOME_FATTY_ACID_METABOLISM | ACOT13 |
| REACTOME_FATTY_ACID_METABOLISM | HSD17B8 |
| REACTOME_FATTY_ACID_METABOLISM | EHHADH |
| REACTOME_FATTY_ACID_METABOLISM | PCCB |
| REACTOME_FATTY_ACID_METABOLISM | ACADL |
| REACTOME_FATTY_ACID_METABOLISM | PECR |
| REACTOME_FATTY_ACID_METABOLISM | SCP2 |
| REACTOME_FATTY_ACID_METABOLISM | MECR |
| REACTOME_FATTY_ACID_METABOLISM | PLA2G4A |
| REACTOME_FATTY_ACID_METABOLISM | HAO2 |
| REACTOME_FATTY_ACID_METABOLISM | ACADM |
| REACTOME_FATTY_ACID_METABOLISM | FAAH |
| REACTOME_FATTY_ACID_METABOLISM | ELOVL4 |
| REACTOME_FATTY_ACID_METABOLISM | ACOT2 |
| REACTOME_FATTY_ACID_METABOLISM | ELOVL3 |
| REACTOME_FATTY_ACID_METABOLISM | EPHX2 |
| REACTOME_FATTY_ACID_METABOLISM | ACADS |
| REACTOME_FATTY_ACID_METABOLISM | ACOT9 |
| REACTOME_FATTY_ACID_METABOLISM | ACSL3 |
| REACTOME_FATTY_ACID_METABOLISM | PTGIS |
| REACTOME_FATTY_ACID_METABOLISM | MCEE |
| REACTOME_FATTY_ACID_METABOLISM | ECHS1 |
| REACTOME_FATTY_ACID_METABOLISM | ACSBG2 |
| REACTOME_FATTY_ACID_METABOLISM | HSD17B3 |
| REACTOME_FATTY_ACID_METABOLISM | PPT1 |
| REACTOME_FATTY_ACID_METABOLISM | HACL1 |
| REACTOME_FATTY_ACID_METABOLISM | ACLY |
| REACTOME_FATTY_ACID_METABOLISM | PRKAB2 |
| REACTOME_FATTY_ACID_METABOLISM | ALOX5AP |
| REACTOME_FATTY_ACID_METABOLISM | MORC2 |
| REACTOME_FATTY_ACID_METABOLISM | HSD17B4 |
| REACTOME_FATTY_ACID_METABOLISM | CYP2J2 |
| REACTOME_FATTY_ACID_METABOLISM | FADS2 |
| REACTOME_FATTY_ACID_METABOLISM | HADHB |
| REACTOME_FATTY_ACID_METABOLISM | CYP1B1 |
| REACTOME_FATTY_ACID_METABOLISM | CYP2C9 |
| REACTOME_FATTY_ACID_METABOLISM | CYP2C8 |
| REACTOME_FATTY_ACID_METABOLISM | HADH |
| REACTOME_FATTY_ACID_METABOLISM | PTGR2 |
| REACTOME_FATTY_ACID_METABOLISM | SLC27A2 |
| REACTOME_FATTY_ACID_METABOLISM | CYP1A1 |
| REACTOME_FATTY_ACID_METABOLISM | CYP1A2 |
| REACTOME_FATTY_ACID_METABOLISM | NUDT7 |
| REACTOME_FATTY_ACID_METABOLISM | DPEP3 |
| REACTOME_FATTY_ACID_METABOLISM | PCTP |
| REACTOME_FATTY_ACID_METABOLISM | CYP4B1 |
| REACTOME_FATTY_ACID_METABOLISM | SLC27A3 |
| REACTOME_FATTY_ACID_METABOLISM | SCD5 |
| REACTOME_FATTY_ACID_METABOLISM | CBR4 |
| REACTOME_FATTY_ACID_METABOLISM | MMUT |
| REACTOME_FATTY_ACID_METABOLISM | PTGES2 |
| REACTOME_FATTY_ACID_METABOLISM | PTGES |
| REACTOME_FATTY_ACID_METABOLISM | HSD17B12 |
| REACTOME_FATTY_ACID_METABOLISM | FADS1 |
| REACTOME_FATTY_ACID_METABOLISM | THRSP |
| REACTOME_FATTY_ACID_METABOLISM | MMAA |
| REACTOME_FATTY_ACID_METABOLISM | ACSL1 |
| REACTOME_FATTY_ACID_METABOLISM | OLAH |
| REACTOME_FATTY_ACID_METABOLISM | ACOXL |
| REACTOME_FATTY_ACID_METABOLISM | CYP2U1 |
| REACTOME_FATTY_ACID_METABOLISM | DBI |
| REACTOME_FATTY_ACID_METABOLISM | CPT2 |
| REACTOME_FATTY_ACID_METABOLISM | PRXL2B |
| REACTOME_FATTY_ACID_METABOLISM | CBR1 |
| REACTOME_FATTY_ACID_METABOLISM | THEM4 |
| REACTOME_FATTY_ACID_METABOLISM | ACOX1 |
| REACTOME_FATTY_ACID_METABOLISM | ALOX15 |
| REACTOME_FATTY_ACID_METABOLISM | CYP4A22 |
| REACTOME_FATTY_ACID_METABOLISM | ACOT11 |
| REACTOME_FATTY_ACID_METABOLISM | PRKAA2 |
| REACTOME_FATTY_ACID_METABOLISM | MAPKAPK2 |
| REACTOME_FATTY_ACID_METABOLISM | HPGDS |
| REACTOME_FATTY_ACID_METABOLISM | HPGD |
| REACTOME_FATTY_ACID_METABOLISM | ELOVL7 |
| REACTOME_FATTY_ACID_METABOLISM | ACSL6 |
| REACTOME_FATTY_ACID_METABOLISM | MID1IP1 |
| REACTOME_FATTY_ACID_METABOLISM | FAAH2 |
| REACTOME_FATTY_ACID_METABOLISM | CYP2C19 |
| REACTOME_FATTY_ACID_METABOLISM | HACD1 |
| REACTOME_FATTY_ACID_METABOLISM | ACSF2 |
| REACTOME_FATTY_ACID_METABOLISM | DPEP2 |
| REACTOME_FATTY_ACID_METABOLISM | ACAA2 |
| REACTOME_FATTY_ACID_METABOLISM | GPX4 |
| REACTOME_FATTY_ACID_METABOLISM | ECI1 |
| REACTOME_FATTY_ACID_METABOLISM | ACOX2 |
| REACTOME_FATTY_ACID_METABOLISM | PPT2 |
| REACTOME_FATTY_ACID_METABOLISM | FASN |
| REACTOME_FATTY_ACID_METABOLISM | ELOVL6 |
| REACTOME_FATTY_ACID_METABOLISM | CYP4F11 |
| REACTOME_FATTY_ACID_METABOLISM | CYP4F22 |
| REACTOME_FATTY_ACID_METABOLISM | ACOT12 |
| REACTOME_FATTY_ACID_METABOLISM | ACSM6 |
| REACTOME_FATTY_ACID_METABOLISM | PCCA |
| REACTOME_FATTY_ACID_METABOLISM | GPX2 |
| REACTOME_FATTY_ACID_METABOLISM | ACBD7 |
| REACTOME_FATTY_ACID_METABOLISM | ACSF3 |
| REACTOME_FATTY_ACID_METABOLISM | ACOT4 |
| REACTOME_FATTY_ACID_METABOLISM | SLC25A20 |
| REACTOME_FATTY_ACID_METABOLISM | ALOXE3 |
| REACTOME_FATTY_ACID_METABOLISM | ALOX12B |
| REACTOME_FATTY_ACID_METABOLISM | ALOX15B |
| REACTOME_FATTY_ACID_METABOLISM | CYP8B1 |
| REACTOME_FATTY_ACID_METABOLISM | ACBD4 |
| REACTOME_FATTY_ACID_METABOLISM | ACOT1 |
| REACTOME_FATTY_ACID_METABOLISM | CYP4F2 |
| REACTOME_FATTY_ACID_METABOLISM | RXRA |
| REACTOME_FATTY_ACID_METABOLISM | CYP4F8 |
| REACTOME_FATTY_ACID_METABOLISM | CYP4F3 |
| REACTOME_FATTY_ACID_METABOLISM | CYP4A11 |
| REACTOME_FATTY_ACID_METABOLISM | HACD4 |
| REACTOME_FATTY_ACID_METABOLISM | AKR1C3 |
| REACTOME_FATTY_ACID_METABOLISM | THEM5 |
| REACTOME_FATTY_ACID_METABOLISM | ACSL5 |
| REACTOME_FATTY_ACID_METABOLISM | SLC22A5 |
| REACTOME_FATTY_ACID_METABOLISM | ELOVL2 |
| REACTOME_FATTY_ACID_METABOLISM | ECI2 |
| REACTOME_FATTY_ACID_METABOLISM | AWAT1 |
| REACTOME_FATTY_ACID_METABOLISM | HSD17B8 |
| REACTOME_FATTY_ACID_METABOLISM | CPT1B |
| REACTOME_FATTY_ACID_METABOLISM | ACOT6 |
| REACTOME_FATTY_ACID_METABOLISM | TECRL |
| REACTOME_FATTY_ACID_METABOLISM | PPT2 |
| REACTOME_FATTY_ACID_METABOLISM | PPT2 |
| REACTOME_FATTY_ACID_METABOLISM | HACD2 |
| REACTOME_FATTY_ACID_METABOLISM | LTC4S |
| REACTOME_FATTY_ACID_METABOLISM | NUDT19 |
| REACTOME_FATTY_ACID_METABOLISM | PPT2 |
| REACTOME_FATTY_ACID_METABOLISM | HSD17B8 |
| REACTOME_FATTY_ACID_METABOLISM | PPT2 |
| REACTOME_FATTY_ACID_METABOLISM | PPT2 |
| REACTOME_FATTY_ACID_METABOLISM | HSD17B8 |
| REACTOME_FATTY_ACID_METABOLISM | HSD17B8 |
| REACTOME_FATTY_ACID_METABOLISM | ACBD6 |
| REACTOME_FATTY_ACID_METABOLISM | PPT2 |
| REACTOME_FATTY_ACID_METABOLISM | HSD17B8 |
| REACTOME_FATTY_ACID_METABOLISM | GPX1 |
| REACTOME_FATTY_ACID_METABOLISM | PPT2 |
| REACTOME_FATTY_ACID_METABOLISM | ACAD11 |
| REACTOME_FATTY_ACID_METABOLISM | AMACR |
| REACTOME_FATTY_ACID_METABOLISM | DECR2 |
| REACTOME_FATTY_ACID_METABOLISM | HTD2 |
| REACTOME_FATTY_ACID_METABOLISM | SLC27A3 |
| REACTOME_FATTY_ACID_METABOLISM | DECR2 |
| REACTOME_FATTY_ACID_METABOLISM | ACACA |
| REACTOME_FATTY_ACID_METABOLISM | ALOX5 |
| REACTOME_FATTY_ACID_METABOLISM | ABCC1 |
| REACTOME_FATTY_ACID_METABOLISM | ACACA |
| REACTOME_FATTY_ACID_METABOLISM | LTC4S |
| REACTOME_FATTY_ACIDS | CYP2D6 |
| REACTOME_FATTY_ACIDS | CYP2J2 |
| REACTOME_FATTY_ACIDS | CYP4B1 |
| REACTOME_FATTY_ACIDS | CYP4A22 |
| REACTOME_FATTY_ACIDS | CYP4F11 |
| REACTOME_FATTY_ACIDS | CYP4F22 |
| REACTOME_FATTY_ACIDS | CYP4F2 |
| REACTOME_FATTY_ACIDS | CYP4F12 |
| REACTOME_FATTY_ACIDS | CYP4F8 |
| REACTOME_FATTY_ACIDS | CYP4F3 |
| REACTOME_FATTY_ACIDS | CYP4A11 |
| REACTOME_FATTY_ACIDS | CYP2B6 |
| REACTOME_FATTY_ACIDS | CYP2F1 |
| REACTOME_FATTY_ACIDS | CYP2A13 |
| REACTOME_FATTY_ACIDS | CYP2A7 |
| REACTOME_FATTY_ACIDS | CYP2D6 |
| REACTOME_FATTY_ACYL_COA_BIOSYNTHESIS | ELOVL5 |
| REACTOME_FATTY_ACYL_COA_BIOSYNTHESIS | ELOVL1 |
| REACTOME_FATTY_ACYL_COA_BIOSYNTHESIS | ACSL4 |
| REACTOME_FATTY_ACYL_COA_BIOSYNTHESIS | HACD3 |
| REACTOME_FATTY_ACYL_COA_BIOSYNTHESIS | SCD |
| REACTOME_FATTY_ACYL_COA_BIOSYNTHESIS | TECR |
| REACTOME_FATTY_ACYL_COA_BIOSYNTHESIS | ACSBG1 |
| REACTOME_FATTY_ACYL_COA_BIOSYNTHESIS | HSD17B8 |
| REACTOME_FATTY_ACYL_COA_BIOSYNTHESIS | ELOVL4 |
| REACTOME_FATTY_ACYL_COA_BIOSYNTHESIS | ELOVL3 |
| REACTOME_FATTY_ACYL_COA_BIOSYNTHESIS | ACSL3 |
| REACTOME_FATTY_ACYL_COA_BIOSYNTHESIS | ACSBG2 |
| REACTOME_FATTY_ACYL_COA_BIOSYNTHESIS | HSD17B3 |
| REACTOME_FATTY_ACYL_COA_BIOSYNTHESIS | PPT1 |
| REACTOME_FATTY_ACYL_COA_BIOSYNTHESIS | ACLY |
| REACTOME_FATTY_ACYL_COA_BIOSYNTHESIS | MORC2 |
| REACTOME_FATTY_ACYL_COA_BIOSYNTHESIS | SLC27A2 |
| REACTOME_FATTY_ACYL_COA_BIOSYNTHESIS | SLC27A3 |
| REACTOME_FATTY_ACYL_COA_BIOSYNTHESIS | SCD5 |
| REACTOME_FATTY_ACYL_COA_BIOSYNTHESIS | CBR4 |
| REACTOME_FATTY_ACYL_COA_BIOSYNTHESIS | HSD17B12 |
| REACTOME_FATTY_ACYL_COA_BIOSYNTHESIS | ACSL1 |
| REACTOME_FATTY_ACYL_COA_BIOSYNTHESIS | OLAH |
| REACTOME_FATTY_ACYL_COA_BIOSYNTHESIS | ELOVL7 |
| REACTOME_FATTY_ACYL_COA_BIOSYNTHESIS | ACSL6 |
| REACTOME_FATTY_ACYL_COA_BIOSYNTHESIS | HACD1 |
| REACTOME_FATTY_ACYL_COA_BIOSYNTHESIS | PPT2 |
| REACTOME_FATTY_ACYL_COA_BIOSYNTHESIS | FASN |
| REACTOME_FATTY_ACYL_COA_BIOSYNTHESIS | ELOVL6 |
| REACTOME_FATTY_ACYL_COA_BIOSYNTHESIS | ACSF3 |
| REACTOME_FATTY_ACYL_COA_BIOSYNTHESIS | HACD4 |
| REACTOME_FATTY_ACYL_COA_BIOSYNTHESIS | ACSL5 |
| REACTOME_FATTY_ACYL_COA_BIOSYNTHESIS | ELOVL2 |
| REACTOME_FATTY_ACYL_COA_BIOSYNTHESIS | HSD17B8 |
| REACTOME_FATTY_ACYL_COA_BIOSYNTHESIS | TECRL |
| REACTOME_FATTY_ACYL_COA_BIOSYNTHESIS | PPT2 |
| REACTOME_FATTY_ACYL_COA_BIOSYNTHESIS | PPT2 |
| REACTOME_FATTY_ACYL_COA_BIOSYNTHESIS | HACD2 |
| REACTOME_FATTY_ACYL_COA_BIOSYNTHESIS | PPT2 |
| REACTOME_FATTY_ACYL_COA_BIOSYNTHESIS | HSD17B8 |
| REACTOME_FATTY_ACYL_COA_BIOSYNTHESIS | PPT2 |
| REACTOME_FATTY_ACYL_COA_BIOSYNTHESIS | PPT2 |
| REACTOME_FATTY_ACYL_COA_BIOSYNTHESIS | HSD17B8 |
| REACTOME_FATTY_ACYL_COA_BIOSYNTHESIS | HSD17B8 |
| REACTOME_FATTY_ACYL_COA_BIOSYNTHESIS | PPT2 |
| REACTOME_FATTY_ACYL_COA_BIOSYNTHESIS | HSD17B8 |
| REACTOME_FATTY_ACYL_COA_BIOSYNTHESIS | PPT2 |
| REACTOME_FATTY_ACYL_COA_BIOSYNTHESIS | HTD2 |
| REACTOME_FATTY_ACYL_COA_BIOSYNTHESIS | SLC27A3 |
| REACTOME_FATTY_ACYL_COA_BIOSYNTHESIS | ACACA |
| REACTOME_FATTY_ACYL_COA_BIOSYNTHESIS | ACACA |
| REACTOME_FREE_FATTY_ACID_RECEPTORS | GPR31 |
| REACTOME_FREE_FATTY_ACID_RECEPTORS | FFAR2 |
| REACTOME_FREE_FATTY_ACID_RECEPTORS | FFAR1 |
| REACTOME_FREE_FATTY_ACID_RECEPTORS | FFAR3 |
| REACTOME_FREE_FATTY_ACID_RECEPTORS | FFAR4 |
| REACTOME_MITOCHONDRIAL_FATTY_ACID_BETA_OXIDATION | NDUFAB1 |
| REACTOME_MITOCHONDRIAL_FATTY_ACID_BETA_OXIDATION | ACSM3 |
| REACTOME_MITOCHONDRIAL_FATTY_ACID_BETA_OXIDATION | ACADVL |
| REACTOME_MITOCHONDRIAL_FATTY_ACID_BETA_OXIDATION | HADHA |
| REACTOME_MITOCHONDRIAL_FATTY_ACID_BETA_OXIDATION | ACOT7 |
| REACTOME_MITOCHONDRIAL_FATTY_ACID_BETA_OXIDATION | MCAT |
| REACTOME_MITOCHONDRIAL_FATTY_ACID_BETA_OXIDATION | DECR1 |
| REACTOME_MITOCHONDRIAL_FATTY_ACID_BETA_OXIDATION | ACAD10 |
| REACTOME_MITOCHONDRIAL_FATTY_ACID_BETA_OXIDATION | ACOT13 |
| REACTOME_MITOCHONDRIAL_FATTY_ACID_BETA_OXIDATION | PCCB |
| REACTOME_MITOCHONDRIAL_FATTY_ACID_BETA_OXIDATION | ACADL |
| REACTOME_MITOCHONDRIAL_FATTY_ACID_BETA_OXIDATION | MECR |
| REACTOME_MITOCHONDRIAL_FATTY_ACID_BETA_OXIDATION | ACADM |
| REACTOME_MITOCHONDRIAL_FATTY_ACID_BETA_OXIDATION | ACOT2 |
| REACTOME_MITOCHONDRIAL_FATTY_ACID_BETA_OXIDATION | ACADS |
| REACTOME_MITOCHONDRIAL_FATTY_ACID_BETA_OXIDATION | ACOT9 |
| REACTOME_MITOCHONDRIAL_FATTY_ACID_BETA_OXIDATION | MCEE |
| REACTOME_MITOCHONDRIAL_FATTY_ACID_BETA_OXIDATION | ECHS1 |
| REACTOME_MITOCHONDRIAL_FATTY_ACID_BETA_OXIDATION | HADHB |
| REACTOME_MITOCHONDRIAL_FATTY_ACID_BETA_OXIDATION | HADH |
| REACTOME_MITOCHONDRIAL_FATTY_ACID_BETA_OXIDATION | PCTP |
| REACTOME_MITOCHONDRIAL_FATTY_ACID_BETA_OXIDATION | MMUT |
| REACTOME_MITOCHONDRIAL_FATTY_ACID_BETA_OXIDATION | MMAA |
| REACTOME_MITOCHONDRIAL_FATTY_ACID_BETA_OXIDATION | DBI |
| REACTOME_MITOCHONDRIAL_FATTY_ACID_BETA_OXIDATION | THEM4 |
| REACTOME_MITOCHONDRIAL_FATTY_ACID_BETA_OXIDATION | ACOT11 |
| REACTOME_MITOCHONDRIAL_FATTY_ACID_BETA_OXIDATION | ACSF2 |
| REACTOME_MITOCHONDRIAL_FATTY_ACID_BETA_OXIDATION | ACAA2 |
| REACTOME_MITOCHONDRIAL_FATTY_ACID_BETA_OXIDATION | ECI1 |
| REACTOME_MITOCHONDRIAL_FATTY_ACID_BETA_OXIDATION | ACOT12 |
| REACTOME_MITOCHONDRIAL_FATTY_ACID_BETA_OXIDATION | ACSM6 |
| REACTOME_MITOCHONDRIAL_FATTY_ACID_BETA_OXIDATION | PCCA |
| REACTOME_MITOCHONDRIAL_FATTY_ACID_BETA_OXIDATION | ACBD7 |
| REACTOME_MITOCHONDRIAL_FATTY_ACID_BETA_OXIDATION | ACOT1 |
| REACTOME_MITOCHONDRIAL_FATTY_ACID_BETA_OXIDATION | THEM5 |
| REACTOME_MITOCHONDRIAL_FATTY_ACID_BETA_OXIDATION | ACBD6 |
| REACTOME_MITOCHONDRIAL_FATTY_ACID_BETA_OXIDATION | ACAD11 |
| REACTOME_MITOCHONDRIAL_FATTY_ACID_BETA_OXIDATION_OF_SATURATED_FATTY_ACIDS | ACSM3 |
| REACTOME_MITOCHONDRIAL_FATTY_ACID_BETA_OXIDATION_OF_SATURATED_FATTY_ACIDS | ACADVL |
| REACTOME_MITOCHONDRIAL_FATTY_ACID_BETA_OXIDATION_OF_SATURATED_FATTY_ACIDS | HADHA |
| REACTOME_MITOCHONDRIAL_FATTY_ACID_BETA_OXIDATION_OF_SATURATED_FATTY_ACIDS | ACADL |
| REACTOME_MITOCHONDRIAL_FATTY_ACID_BETA_OXIDATION_OF_SATURATED_FATTY_ACIDS | MECR |
| REACTOME_MITOCHONDRIAL_FATTY_ACID_BETA_OXIDATION_OF_SATURATED_FATTY_ACIDS | ACADM |
| REACTOME_MITOCHONDRIAL_FATTY_ACID_BETA_OXIDATION_OF_SATURATED_FATTY_ACIDS | ACADS |
| REACTOME_MITOCHONDRIAL_FATTY_ACID_BETA_OXIDATION_OF_SATURATED_FATTY_ACIDS | ECHS1 |
| REACTOME_MITOCHONDRIAL_FATTY_ACID_BETA_OXIDATION_OF_SATURATED_FATTY_ACIDS | HADHB |
| REACTOME_MITOCHONDRIAL_FATTY_ACID_BETA_OXIDATION_OF_SATURATED_FATTY_ACIDS | HADH |
| REACTOME_MITOCHONDRIAL_FATTY_ACID_BETA_OXIDATION_OF_SATURATED_FATTY_ACIDS | ACSM6 |
| REACTOME_MITOCHONDRIAL_FATTY_ACID_BETA_OXIDATION_OF_UNSATURATED_FATTY_ACIDS | HADHA |
| REACTOME_MITOCHONDRIAL_FATTY_ACID_BETA_OXIDATION_OF_UNSATURATED_FATTY_ACIDS | DECR1 |
| REACTOME_MITOCHONDRIAL_FATTY_ACID_BETA_OXIDATION_OF_UNSATURATED_FATTY_ACIDS | ACADL |
| REACTOME_MITOCHONDRIAL_FATTY_ACID_BETA_OXIDATION_OF_UNSATURATED_FATTY_ACIDS | ACADM |
| REACTOME_MITOCHONDRIAL_FATTY_ACID_BETA_OXIDATION_OF_UNSATURATED_FATTY_ACIDS | HADHB |
| REACTOME_MITOCHONDRIAL_FATTY_ACID_BETA_OXIDATION_OF_UNSATURATED_FATTY_ACIDS | ECI1 |
| REACTOME_SYNTHESIS_OF_VERY_LONG_CHAIN_FATTY_ACYL_COAS | ELOVL5 |
| REACTOME_SYNTHESIS_OF_VERY_LONG_CHAIN_FATTY_ACYL_COAS | ELOVL1 |
| REACTOME_SYNTHESIS_OF_VERY_LONG_CHAIN_FATTY_ACYL_COAS | ACSL4 |
| REACTOME_SYNTHESIS_OF_VERY_LONG_CHAIN_FATTY_ACYL_COAS | HACD3 |
| REACTOME_SYNTHESIS_OF_VERY_LONG_CHAIN_FATTY_ACYL_COAS | TECR |
| REACTOME_SYNTHESIS_OF_VERY_LONG_CHAIN_FATTY_ACYL_COAS | ACSBG1 |
| REACTOME_SYNTHESIS_OF_VERY_LONG_CHAIN_FATTY_ACYL_COAS | ELOVL4 |
| REACTOME_SYNTHESIS_OF_VERY_LONG_CHAIN_FATTY_ACYL_COAS | ELOVL3 |
| REACTOME_SYNTHESIS_OF_VERY_LONG_CHAIN_FATTY_ACYL_COAS | ACSL3 |
| REACTOME_SYNTHESIS_OF_VERY_LONG_CHAIN_FATTY_ACYL_COAS | ACSBG2 |
| REACTOME_SYNTHESIS_OF_VERY_LONG_CHAIN_FATTY_ACYL_COAS | HSD17B3 |
| REACTOME_SYNTHESIS_OF_VERY_LONG_CHAIN_FATTY_ACYL_COAS | SLC27A3 |
| REACTOME_SYNTHESIS_OF_VERY_LONG_CHAIN_FATTY_ACYL_COAS | HSD17B12 |
| REACTOME_SYNTHESIS_OF_VERY_LONG_CHAIN_FATTY_ACYL_COAS | ACSL1 |
| REACTOME_SYNTHESIS_OF_VERY_LONG_CHAIN_FATTY_ACYL_COAS | ELOVL7 |
| REACTOME_SYNTHESIS_OF_VERY_LONG_CHAIN_FATTY_ACYL_COAS | ACSL6 |
| REACTOME_SYNTHESIS_OF_VERY_LONG_CHAIN_FATTY_ACYL_COAS | HACD1 |
| REACTOME_SYNTHESIS_OF_VERY_LONG_CHAIN_FATTY_ACYL_COAS | ELOVL6 |
| REACTOME_SYNTHESIS_OF_VERY_LONG_CHAIN_FATTY_ACYL_COAS | ACSF3 |
| REACTOME_SYNTHESIS_OF_VERY_LONG_CHAIN_FATTY_ACYL_COAS | HACD4 |
| REACTOME_SYNTHESIS_OF_VERY_LONG_CHAIN_FATTY_ACYL_COAS | ACSL5 |
| REACTOME_SYNTHESIS_OF_VERY_LONG_CHAIN_FATTY_ACYL_COAS | ELOVL2 |
| REACTOME_SYNTHESIS_OF_VERY_LONG_CHAIN_FATTY_ACYL_COAS | TECRL |
| REACTOME_SYNTHESIS_OF_VERY_LONG_CHAIN_FATTY_ACYL_COAS | HACD2 |
| REACTOME_SYNTHESIS_OF_VERY_LONG_CHAIN_FATTY_ACYL_COAS | SLC27A3 |
| REACTOME_THE_FATTY_ACID_CYCLING_MODEL | SLC25A14 |
| REACTOME_THE_FATTY_ACID_CYCLING_MODEL | UCP1 |
| REACTOME_THE_FATTY_ACID_CYCLING_MODEL | SLC25A27 |
| REACTOME_THE_FATTY_ACID_CYCLING_MODEL | UCP3 |
| REACTOME_THE_FATTY_ACID_CYCLING_MODEL | UCP2 |
| REACTOME_TRANSPORT_OF_FATTY_ACIDS | SLC27A6 |
| REACTOME_TRANSPORT_OF_FATTY_ACIDS | SLC27A1 |
| REACTOME_TRANSPORT_OF_FATTY_ACIDS | LCN9 |
| REACTOME_TRANSPORT_OF_FATTY_ACIDS | LCN1 |
| REACTOME_TRANSPORT_OF_FATTY_ACIDS | SLC27A4 |
| REACTOME_TRANSPORT_OF_FATTY_ACIDS | LCN15 |
| REACTOME_TRANSPORT_OF_FATTY_ACIDS | LCN12 |
| REACTOME_TRANSPORT_OF_FATTY_ACIDS | APOD |
