# Supplementary material for: Robust machine-learning based prognostic index using fatty acid metabolism genes predicts prognosis and therapy responses in glioblastoma
Source: J Cancer. 2025 Aug 22;16(13):3859–73. doi: 10.7150/jca.117209 (PMC12490975; doi:10.7150/jca.117209)
Supplement: Supplementary file 1 — Supplementary figures and tables. [file jcav16p3859s1.zip › Supplemental TableS2.docx]

**Table S2. The Consensus clustering result of GBM samples**

| **Barcode** | **Cluster** |
| --- | --- |
| TCGA-02-0047-01 | Cluster1 |
| TCGA-02-0055-01 | Cluster1 |
| TCGA-02-2483-01 | Cluster2 |
| TCGA-02-2485-01 | Cluster2 |
| TCGA-02-2486-01 | Cluster1 |
| TCGA-06-0125-01 | Cluster2 |
| TCGA-06-0125-02 | Cluster1 |
| TCGA-06-0129-01 | Cluster2 |
| TCGA-06-0130-01 | Cluster1 |
| TCGA-06-0132-01 | Cluster1 |
| TCGA-06-0138-01 | Cluster1 |
| TCGA-06-0141-01 | Cluster1 |
| TCGA-06-0152-02 | Cluster1 |
| TCGA-06-0156-01 | Cluster1 |
| TCGA-06-0157-01 | Cluster2 |
| TCGA-06-0158-01 | Cluster1 |
| TCGA-06-0168-01 | Cluster1 |
| TCGA-06-0171-02 | Cluster1 |
| TCGA-06-0174-01 | Cluster2 |
| TCGA-06-0178-01 | Cluster2 |
| TCGA-06-0184-01 | Cluster2 |
| TCGA-06-0187-01 | Cluster2 |
| TCGA-06-0190-01 | Cluster1 |
| TCGA-06-0190-02 | Cluster1 |
| TCGA-06-0210-01 | Cluster1 |
| TCGA-06-0210-02 | Cluster1 |
| TCGA-06-0211-01 | Cluster2 |
| TCGA-06-0211-02 | Cluster1 |
| TCGA-06-0219-01 | Cluster2 |
| TCGA-06-0221-02 | Cluster2 |
| TCGA-06-0238-01 | Cluster2 |
| TCGA-06-0644-01 | Cluster1 |
| TCGA-06-0645-01 | Cluster1 |
| TCGA-06-0646-01 | Cluster1 |
| TCGA-06-0649-01 | Cluster1 |
| TCGA-06-0686-01 | Cluster1 |
| TCGA-06-0743-01 | Cluster1 |
| TCGA-06-0744-01 | Cluster2 |
| TCGA-06-0745-01 | Cluster1 |
| TCGA-06-0747-01 | Cluster2 |
| TCGA-06-0749-01 | Cluster1 |
| TCGA-06-0750-01 | Cluster1 |
| TCGA-06-0878-01 | Cluster1 |
| TCGA-06-0882-01 | Cluster1 |
| TCGA-06-1804-01 | Cluster2 |
| TCGA-06-2557-01 | Cluster1 |
| TCGA-06-2558-01 | Cluster2 |
| TCGA-06-2559-01 | Cluster2 |
| TCGA-06-2561-01 | Cluster1 |
| TCGA-06-2562-01 | Cluster2 |
| TCGA-06-2563-01 | Cluster2 |
| TCGA-06-2564-01 | Cluster2 |
| TCGA-06-2565-01 | Cluster2 |
| TCGA-06-2567-01 | Cluster2 |
| TCGA-06-2569-01 | Cluster2 |
| TCGA-06-2570-01 | Cluster2 |
| TCGA-06-5408-01 | Cluster1 |
| TCGA-06-5410-01 | Cluster1 |
| TCGA-06-5411-01 | Cluster1 |
| TCGA-06-5412-01 | Cluster1 |
| TCGA-06-5413-01 | Cluster2 |
| TCGA-06-5414-01 | Cluster2 |
| TCGA-06-5415-01 | Cluster2 |
| TCGA-06-5416-01 | Cluster2 |
| TCGA-06-5417-01 | Cluster2 |
| TCGA-06-5418-01 | Cluster2 |
| TCGA-06-5856-01 | Cluster2 |
| TCGA-06-5858-01 | Cluster2 |
| TCGA-06-5859-01 | Cluster1 |
| TCGA-08-0386-01 | Cluster2 |
| TCGA-12-0616-01 | Cluster2 |
| TCGA-12-0618-01 | Cluster1 |
| TCGA-12-0619-01 | Cluster1 |
| TCGA-12-0821-01 | Cluster2 |
| TCGA-12-1597-01 | Cluster2 |
| TCGA-12-3650-01 | Cluster2 |
| TCGA-12-3652-01 | Cluster2 |
| TCGA-12-3653-01 | Cluster2 |
| TCGA-12-5295-01 | Cluster2 |
| TCGA-12-5299-01 | Cluster2 |
| TCGA-14-0736-02 | Cluster1 |
| TCGA-14-0781-01 | Cluster1 |
| TCGA-14-0787-01 | Cluster2 |
| TCGA-14-0789-01 | Cluster1 |
| TCGA-14-0790-01 | Cluster2 |
| TCGA-14-0817-01 | Cluster2 |
| TCGA-14-0871-01 | Cluster2 |
| TCGA-14-1034-01 | Cluster1 |
| TCGA-14-1034-02 | Cluster2 |
| TCGA-14-1402-02 | Cluster2 |
| TCGA-14-1823-01 | Cluster2 |
| TCGA-14-1825-01 | Cluster2 |
| TCGA-14-1829-01 | Cluster2 |
| TCGA-14-2554-01 | Cluster2 |
| TCGA-15-0742-01 | Cluster2 |
| TCGA-15-1444-01 | Cluster2 |
| TCGA-16-0846-01 | Cluster1 |
| TCGA-16-1045-01 | Cluster1 |
| TCGA-19-0957-02 | Cluster1 |
| TCGA-19-1389-02 | Cluster1 |
| TCGA-19-1390-01 | Cluster1 |
| TCGA-19-1787-01 | Cluster1 |
| TCGA-19-2619-01 | Cluster2 |
| TCGA-19-2620-01 | Cluster1 |
| TCGA-19-2624-01 | Cluster2 |
| TCGA-19-2625-01 | Cluster1 |
| TCGA-19-2629-01 | Cluster2 |
| TCGA-19-4065-01 | Cluster1 |
| TCGA-19-4065-02 | Cluster1 |
| TCGA-19-5960-01 | Cluster2 |
| TCGA-26-1442-01 | Cluster2 |
| TCGA-26-5132-01 | Cluster2 |
| TCGA-26-5133-01 | Cluster2 |
| TCGA-26-5134-01 | Cluster1 |
| TCGA-26-5135-01 | Cluster1 |
| TCGA-26-5136-01 | Cluster1 |
| TCGA-26-5139-01 | Cluster2 |
| TCGA-27-1830-01 | Cluster1 |
| TCGA-27-1831-01 | Cluster2 |
| TCGA-27-1832-01 | Cluster1 |
| TCGA-27-1834-01 | Cluster1 |
| TCGA-27-1835-01 | Cluster2 |
| TCGA-27-1837-01 | Cluster2 |
| TCGA-27-2519-01 | Cluster1 |
| TCGA-27-2521-01 | Cluster2 |
| TCGA-27-2523-01 | Cluster2 |
| TCGA-27-2524-01 | Cluster1 |
| TCGA-27-2526-01 | Cluster2 |
| TCGA-27-2528-01 | Cluster2 |
| TCGA-28-1747-01 | Cluster2 |
| TCGA-28-1753-01 | Cluster1 |
| TCGA-28-2499-01 | Cluster2 |
| TCGA-28-2509-01 | Cluster1 |
| TCGA-28-2513-01 | Cluster1 |
| TCGA-28-2514-01 | Cluster1 |
| TCGA-28-5204-01 | Cluster2 |
| TCGA-28-5207-01 | Cluster2 |
| TCGA-28-5208-01 | Cluster2 |
| TCGA-28-5209-01 | Cluster2 |
| TCGA-28-5213-01 | Cluster1 |
| TCGA-28-5215-01 | Cluster1 |
| TCGA-28-5216-01 | Cluster1 |
| TCGA-28-5218-01 | Cluster1 |
| TCGA-28-5220-01 | Cluster2 |
| TCGA-32-1970-01 | Cluster2 |
| TCGA-32-1980-01 | Cluster1 |
| TCGA-32-1982-01 | Cluster2 |
| TCGA-32-2615-01 | Cluster1 |
| TCGA-32-2616-01 | Cluster1 |
| TCGA-32-2632-01 | Cluster2 |
| TCGA-32-2634-01 | Cluster1 |
| TCGA-32-2638-01 | Cluster1 |
| TCGA-32-4213-01 | Cluster1 |
| TCGA-32-5222-01 | Cluster2 |
| TCGA-41-2571-01 | Cluster1 |
| TCGA-41-2572-01 | Cluster2 |
| TCGA-41-3915-01 | Cluster1 |
| TCGA-41-4097-01 | Cluster1 |
| TCGA-41-5651-01 | Cluster2 |
| TCGA-76-4925-01 | Cluster2 |
| TCGA-76-4926-01 | Cluster2 |
| TCGA-76-4927-01 | Cluster2 |
| TCGA-76-4928-01 | Cluster1 |
| TCGA-76-4929-01 | Cluster1 |
| TCGA-76-4931-01 | Cluster2 |
| TCGA-76-4932-01 | Cluster2 |
