# Supplementary material for: Robust machine-learning based prognostic index using fatty acid metabolism genes predicts prognosis and therapy responses in glioblastoma
Source: J Cancer. 2025 Aug 22;16(13):3859–73. doi: 10.7150/jca.117209 (PMC12490975; doi:10.7150/jca.117209)
Supplement: Supplementary file 1 — Supplementary figures and tables. [file jcav16p3859s1.zip › Supplemental TableS3.docx]

**Table S3. The wald scores of each gene computed by superPC and description of fatty acid signature genes.**

| **Gene** | **Gene name** | **Wald Score** |
| --- | --- | --- |
| G0S2 | G0/G1 switch protein 2 | 1.771195 |
| LDHA | Lactate dehydrogenase A | 1.222994 |
| ACOT7 | Cytosolic acyl coenzyme A thioester hydrolase | 0.950138 |
| ADH1C | Alcohol dehydrogenase 1C | 0.666446 |
| ADH1A | Alcohol dehydrogenase 1A | 0.823875 |
| APEX1 | DNA repair nuclease/redox regulator APEX1 | -1.33836 |
| CBR1 | Carbonyl reductase [NADPH] 1 | 1.165048 |
| NBN | Nibrin | -1.00085 |
| CD1D | Antigen-presenting glycoprotein CD1d | 1.082245 |
| GPX2 | Glutathione peroxidase 2 | 1.244164 |
